# Novel Frailty Assessment Based on Multidimensional Physical Frailty Parameters Using Unsupervised Clustering in Respiratory Diseases: A Pilot Study

**DOI:** 10.3390/jcm15135145

**Published:** 2026-07-01

**Authors:** Keiko Doi, Yoshiyuki Asai, Tsunahiko Hirano, Keiji Oishi, Ayumi Fukatsu-Chikumoto, Tasuku Yamamoto, Yoriyuki Murata, Yuichi Ohteru, Kazuki Hamada, Maki Asami-Noyama, Nobutaka Edakuni, Toshiaki Utsunomiya, Tomoyuki Kakugawa, Kazuto Matsunaga

**Affiliations:** 1 Department of Respiratory Medicine and Infectious Disease, Graduate School of Medicine, Yamaguchi University, 1-1-1 Minami-Kogushi, Ube 755-8505, Japan; decem119@yamaguchi-u.ac.jp (K.D.); ohishk@yamaguchi-u.ac.jp (K.O.); chiku05@yamaguchi-u.ac.jp (A.F.-C.); t.yama93@yamaguchi-u.ac.jp (T.Y.); yomurata@yamaguchi-u.ac.jp (Y.M.); teru1153@yahoo.co.jp (Y.O.); khamada@yamaguchi-u.ac.jp (K.H.); noyamama@yamaguchi-u.ac.jp (M.A.-N.); edakuni@yamaguchi-u.ac.jp (N.E.); t_utsunomiya0316@yahoo.co.jp (T.U.); kazmatsu@yamaguchi-u.ac.jp (K.M.); 2 Department of Pulmonology and Gerontology, Graduate School of Medicine, Yamaguchi University, 1-1-1 Minami-Kogushi, Ube 755-8505, Japan; kakugawa@yamaguchi-u.ac.jp; 3 Department of Systems Bioinformatics, Graduate School of Medicine, Yamaguchi University, 1-1-1 Minami-Kogushi, Ube 755-8505, Japan; asai@yamaguchi-u.ac.jp; 4 AI Systems Medicine Research and Training Center, Graduate School of Medicine, Yamaguchi University, Yamaguchi University Hospital, 1-1-1 Minami-Kogushi, Ube 755-8505, Japan; 5 The Division of Systems Medicine and Informatics, Research Institute for Cell Design Medical Science, Yamaguchi University, 1-1-1 Minami-Kogushi, Ube 755-8505, Japan

**Keywords:** frailty, machine learning, respiratory disease

## Abstract

**Background:** Frailty impacts the prognosis of respiratory diseases but lacks standardized evaluation criteria. This pilot study aimed to develop a frailty assessment method using unsupervised clustering of various physical function tests. **Methods:** Clinical data, handgrip strength (HS), lower limb strength (LLS), the 6 min walk test (6 min WT), the 5 m walk test (5 m WT), body composition, such as skeletal muscle mass index (SMI), whole-body phase angle (WBPhA), and pulmonary function variables were measured. Frailty status was evaluated in three groups (frail, pre-frail, and robust) using the J-CHS and Kihon Checklist. Unsupervised hierarchical clustering was performed, followed by dimensionality reduction using Principal Component Analysis. **Results:** Ninety-eight patients and healthy volunteers (70 males, 28 females; mean age, 57.5 years) were divided into four clusters, ranging from robust to pronounced frailty. On the 2-principal component plane, data points formed clusters across the four regions. The biplot showed variables aggregating in two directions: one including %FEV_1_, FEV_1_%, 6 min WT, and 5 m WT speed (exercise tolerance), and the other including HS, LLS, SMI, and WBPhA (physical elements). Tracking 39 participants (mean, 636 days later) showed cluster shifts that were broadly reproducible, although the small follow-up sample warrants cautious interpretation. **Conclusions:** As an exploratory, hypothesis-generating pilot study with a small single-center sample, this novel frailty model may offer a more granular assessment to help guide management; however, external validation in larger cohorts is required before clinical application.

## 1. Introduction

Frailty is defined as a state in which decreased physiological reserve in old age increases vulnerability to stress, potentially leading to outcomes such as functional impairment, long-term care needs, and death [1,2]. A high prevalence of frailty has been reported in older adult patients with respiratory diseases, including chronic obstructive pulmonary disease (COPD) [3,4,5,6]. Frailty is associated with lower quality of life and poorer prognosis, making it an important therapeutic target. Therefore, the early and accurate diagnosis and management of this condition are crucial. International respiratory societies, including the American Thoracic Society and the European Respiratory Society, have also emphasized the assessment and management of physical impairment and frailty in chronic respiratory disease [7,8].

Frailty encompasses diverse conditions, including physical, mental, and psychological issues, such as cognitive impairment and depression, as well as social problems, such as living alone and economic hardships [2,9,10]. Uniform evaluation of frailty is challenging because of its complex manifestations. Internationally, the Cardiovascular Health Study (CHS) criteria and the Frailty Index are representative assessment methods. In Japan, various methods, such as the CHS and Kihon Checklist (KCL), are used to identify older adults at risk of needing support and care. However, frailty lacks a precise global classification. The limitations of current methods are evident in their categorical nature, which does not capture the continuum of frailty. This underscores the need for an objective, data-driven assessment tool that can provide more nuanced insights into frailty. Machine learning models have the potential to address the limitations of frailty assessment by using continuous variables and enable a more granular frailty classification. Unsupervised learning, which does not require labeled data, enables the construction of an objective model using the characteristics of the data.

Machine learning has shown promise in various areas of healthcare, such as predictive diagnostics and personalized treatment plans [11]. Its ability to handle large datasets and uncover complex patterns makes it particularly suitable for assessing multifactorial conditions such as frailty [12]. The decline in physical ability in respiratory patients could be related to frailty, with contributing factors including decreased muscle strength, walking ability, and body composition [5]. We therefore hypothesized that this technique could provide a model for the objective evaluation of this condition. In this study, we aimed to accurately assess frailty as a continuous variable by using these factors and unsupervised clustering methods.

## 2. Materials and Methods

### 2.1. Study Participants

We recruited participants with or without pulmonary diseases who were undergoing treatment at the Department of Respiratory and Infectious Diseases, Yamaguchi University Hospital, as well as healthy volunteers, including Yamaguchi University medical students. Recruitment occurred from August 2019 to March 2021 for baseline measurements and from June 2021 to July 2022 for follow-up measurements. All participants were older than 20 years. The study protocol and amendments were approved by the local ethics committee of Yamaguchi University (institutional review board no. H2019-030 and 2021-031). Patients who did not complete the study assessments were excluded. All participants received an explanation of the study protocol and provided written informed consent.

### 2.2. Data Collection

Clinical data, including disease (COPD or non-COPD), sex, age, and smoking status (current, ex, ir non-smoker), were extracted from the patients’ medical records. For healthy volunteers, the same information was obtained through direct interviews. Participants were categorized into three groups: “COPD,” “non-COPD,” and “healthy subjects.” COPD diagnosis followed the Japanese Respiratory Society Guidelines (Guidelines for COPD Diagnosis and Treatment 2018 5th edition [13]), requiring a post-bronchodilator FEV_1_% < 70%. Respiratory diseases other than COPD (asthma, bronchiectasis, and chronic respiratory tract infection) were classified as non-COPD. Patients with respiratory diseases were stable, with no exacerbations for at least four weeks prior to the study and had no injuries or lower limb paralysis. Healthy subjects included patients without respiratory diseases and medical students, all of whom had no injuries or leg paralysis. COPD severity was characterized by the spirometric parameters %FEV_1_ and FEV_1_%, which were included as continuous variables in the clustering; discrete severity (GOLD) stages [14] were not used as separate input variables.

The measurements were performed twice. Baseline measurements included 98 participants. Follow-up measurements were conducted with 39 participants. The interval between the first and second measurements had a mean of 636 days with a standard deviation of ±151 days. The items measured were as follows.

#### 2.2.1. Assessment of Pulmonary Function

According to the recommendations of the American Thoracic Society and the European Respiratory Society [15], pulmonary function was assessed using a multifunctional spirometer, the HI-801 (Chest Ltd., Tokyo, Japan). The Japanese reference values for pulmonary function were used [16].

#### 2.2.2. Six-Minute Walk Test

The 6 min WT was performed according to the American Thoracic Society Guidelines. The subjects were instructed to walk as far as possible along a straight, flat course marked at 1 m intervals. The total walking distance was visually measured, and walking speed was calculated by dividing this distance by the walking time.

#### 2.2.3. Five-Meter Walk Test

The 5 m WT was conducted in accordance with the Japanese Society of Physical Therapy guidelines. A 5 m walking path was set up with 3 m auxiliary paths before and after the main path. Participants started walking 3 m before the main path and continued until both feet crossed the end of the main path. The time spent walking along the main path was measured using a stopwatch.

#### 2.2.4. Handgrip Strength

HS was measured using a Digital Grip Dynamometer (TAKEI SCIENTIFIC INSTRUMENTS Co. Ltd., Niigata, Japan). The participants were instructed to stand upright, let their arms hang naturally, and squeeze the grip with full force. Two readings were obtained from each hand, and the higher score was used as the measured HS value for each hand [17].

#### 2.2.5. Lower Limb Strength

LLS was measured using a handheld dynamometer (μTas F-1; Anima Corp., Tokyo, Japan) in a sitting position, with a belt attached to the bedpost and ankle. The participants were positioned with the hip and knee joints at 90° flexion and instructed to extend the knee joint with maximal effort. Measurements were performed three times on each side.

#### 2.2.6. Body Composition

Body composition parameters, such as SMI and WBPhA, were measured by bioelectrical impedance analysis [18] using the InBody S10 (InBody Japan Inc., Tokyo, Japan) in the supine position. SMI was calculated by dividing the mass of the limb skeletal muscle (kg) by the square of the height (m^2^), while WBPhA reflects cell membrane integrity and cellular health.

#### 2.2.7. Frailty Status

Frailty status was evaluated using three methods: the J-CHS (before the 2020 revision) [1,19,20], the KCL [21,22], and the Physician Classification. The J-CHS assesses frailty status based on five elements of Fried’s frailty phenotype: shrinking, exhaustion, low levels of activity, weakness, and slowness. Participants were classified as robust (0), pre-frail (1–2), or frail (3–5) based on these criteria. The KCL is a 25-item questionnaire covering instrumental and social activities of daily living, physical function, nutritional status, oral function, cognitive function, and depressive mood. It was developed by the Ministry of Health, Labor, and Welfare for nursing care prevention. If the score was 0–3, the participant was categorized as robust; 4–7, as pre-frail; and 8–25, as frail. Physician Classification was performed by a single respiratory physician with more than 20 years of experience, based on the participants’ overall health status, serving as supplementary data and enabling a comparison between physician assessments and clustering-based evaluations. Because the J-CHS, KCL, and Physician Classification capture conceptually different aspects of frailty (physical phenotype, a multidomain questionnaire, and holistic clinical judgment, respectively), no single instrument was treated as an absolute gold standard. The Physician Classification was regarded as the most clinically integrative reference but was used as supplementary data given that assessments were made by only a single physician. Agreement among the three labels was quantified using the ARI rather than by forcing concordance, and discordant cases were retained and examined rather than excluded.

### 2.3. Data Preprocessing

The preprocessing of the data involved two steps to prepare for hierarchical clustering and dimensionality reduction by PCA. All clustering inputs were based on complete-case data: participants with any missing value among the analyzed variables were excluded, leaving 98 of the 119 recruited participants for analysis (Figure S1). No imputation was performed, ensuring that all variables entered into the clustering were directly measured rather than estimated. Because individual-level data for the 21 excluded participants were not available, a formal comparison between included and excluded participants could not be undertaken.

1. Standardization: All continuous variables measured during baseline data collection were standardized by subtracting the mean and dividing by the standard deviation, resulting in features with a mean of zero and a standard deviation of one. For the follow-up data, the same parameters from the baseline measurements were used to ensure consistency and comparability.
2. One-hot Encoding: Categorical variables, including smoking history and disease history, were transformed into dummy variables using one-hot encoding, resulting in six additional dummy variables. The same encoding approach used in the initial measurement was applied to the follow-up data to maintain data integrity.

### 2.4. Hierarchical Clustering

Hierarchical clustering was conducted on the dataset comprising 11 standardized parameters and 6 dummy variables from the baseline data collection. We employed the Ward method, which minimizes the sum of squared differences within all clusters, using the Euclidean distance metric to measure dissimilarity between data points. This analytical approach was selected because of its ability to effectively group data points, optimize variance, and ensure cohesive clusters.

The Ward method was selected over alternative approaches for reasons confirmed empirically (Tables S1–S3; Figure S2). Because frailty in this cohort formed a continuous spectrum rather than well-separated compact groups, methods assuming specific cluster geometries performed poorly. Density-based clustering (DBSCAN) recovered no meaningful partition across a range of neighborhood radii, assigning the data to a single cluster with residual noise, consistent with the absence of density gaps (Table S3). k-means (k = 4) was unstable: across 50 random initializations (each with 10 restarts), it converged to 26 distinct local optima, with a mean pairwise ARI = 0.81 and identical solutions in only 3.8% of comparisons; with single initialization, the instability was greater (mean pairwise ARI = 0.60) (Figure S2; Table S2). A Gaussian mixture model (k = 4) likewise diverged from the hierarchical solution (ARI = 0.37; Table S1). In contrast, Ward’s hierarchical clustering is fully deterministic, producing an identical solution on repeated runs, and yields an interpretable nested dendrogram structure in which the three-cluster solution is progressively subdivided into four clusters (Section 3.4; cophenetic correlation, r = 0.59). Notably, the majority-vote consensus of the 50 k-means solutions agreed with the Ward partition (ARI = 0.57), indicating that the Ward solution is consistent with the central tendency of repeated partitions rather than being arbitrary. Determinism, reproducibility, and an interpretable hierarchical structure—properties essential for a clinically applicable assessment tool—motivated the choice of Ward’s method.

After standardizing the follow-up data according to the parameters from the baseline data collection, each data point was assigned to the nearest existing cluster. This allocation was performed using a nearest centroid algorithm, which computes the Euclidean distance between each new data point and the centroid of each previously established cluster and assigns each point to the nearest centroid.

The number of clusters was examined using internal validity indices for k = 2 to 7 (silhouette, Davies–Bouldin, Calinski–Harabasz, and the gap statistic with its standard error [23]) and the cophenetic correlation. Because these indices presuppose well-separated discrete clusters, partition reproducibility was additionally assessed without assuming a single optimal k, using bootstrap cluster stability (Jaccard coefficient, B = 500) [24] and consensus clustering (500 subsamples at 80% sampling, summarized by PAC and a per-subject consensus membership score) [25].

### 2.5. PCA

PCA [26] was conducted on the standardized data to reduce dimensionality. The first two principal components, which accounted for the highest variance, were used to create a two-dimensional scatter plot. This visualization helped illustrate the distribution of and relationships among the data points, enhancing our understanding of the underlying patterns.

Additionally, a biplot analysis was performed by superimposing the loading plots on the scatter plot of the projected data points in the reduced two-dimensional plane. By combining the scores (data points) and loadings (variables), a biplot provides insights into how the original variables relate to the PCs and helps interpret the directions and magnitudes of the variables’ contributions to the data structure.

Data pre-processing and all other analyses were conducted using Python 3.11.5. Specifically, for PCA, hierarchical clustering, and Adjusted Rand Index (ARI) analysis, the scikit-learn library was used.

### 2.6. Statistical Characterization of Clusters

Differences across clusters were quantified for each of the eleven continuous physical function variables. For each variable, a one-way ANOVA was performed, and the effect size was reported as η^2^ with its 95% confidence interval (derived from the noncentral F distribution) and as the bias-corrected ω^2^; Kruskal–Wallis tests with ε^2^ were computed as nonparametric confirmation. ANOVA *p* values across variables were corrected using the Benjamini–Hochberg procedure. All pairwise differences between the four clusters (six comparisons per variable, 66 in total) were assessed using Tukey’s HSD, reporting the mean difference with its 95% CI and the family-wise-corrected *p* value, together with Hedges’ g (small-sample-corrected standardized mean difference) and its 95% CI. To verify robustness to unequal variances across clusters, Games–Howell tests were performed as a sensitivity analysis. The complete pairwise results are provided in Supplementary Table S4, the Games–Howell sensitivity analysis in Supplementary Table S5, and a per-variable summary in Supplementary Table S6.

### 2.7. Sensitivity Analyses

Four sensitivity analyses assessed the robustness of the four-cluster solution. First, internal validity indices (silhouette, Davies–Bouldin, Calinski–Harabasz, and the gap statistic with its standard error), bootstrap cluster stability (Jaccard coefficient, B = 500), and the cophenetic correlation were computed across k = 2–7, and consensus clustering (Monti et al.; 500 subsamples at 80% sampling), summarized by the proportion of ambiguous clustering (PAC) and a per-subject consensus membership score, was performed for k = 3 and k = 4 (Figures S3–S5, Tables S7 and S8). Second, to assess dependence on the categorical inputs, hierarchical clustering was repeated after removing the six disease/smoking dummy variables, using only the eleven standardized continuous variables. Agreement with the original four-cluster solution was quantified using the ARI, and the resulting clusters were examined to confirm preservation of the robust-to-frail gradient (Figures S6 and S7; Table S9). Third, age- and sex-adjusted analyses evaluated potential confounding (Tables S10–S14; Figures S8 and S9). Fourth, all between-cluster comparisons were corroborated the variance-robust Games–Howell test (Table S5).

Adjustment for age and sex. To assess whether the identified cluster structure was confounded by age and sex, we performed three complementary analyses. First, the distribution of age (one-way ANOVA and Kruskal–Wallis test) and sex (chi-squared test) across clusters were examined. Second, an ordinal logistic regression was fitted with cluster membership (ordered from High Function to Severe Impairment) as the outcome and the first two principal components (PC1, PC2; each standardized to 1 SD), age, and sex as predictors; PC1 and PC2 jointly summarize the eleven standardized physical function variables. Third, for each physical function variable, a linear model (variable ~ cluster + age + sex) was fitted to obtain age- and sex-adjusted cluster effects, with *p* values corrected for multiple comparisons using the Benjamini–Hochberg procedure. To distinguish a genuine absence of an independent age effect from a statistical artifact of collinearity, we additionally quantified the Pearson correlation between age and the principal component scores (age treated as a supplementary variable, as it was not included in the clustering or PCA), computed variance inflation factors (VIFs) for the regression predictors, and compared the McFadden pseudo-R^2^ of nested ordinal models. Analyses were implemented in Python using statsmodels.

### 2.8. Statistical Software

Statistical analyses and visualizations were performed using Python 3.11.5 with the following libraries: scikit-learn 1.8.0 (clustering, PCA), statsmodels 0.14.6 (multiple comparisons), scipy 1.17.1, numpy 2.3.5, pandas 3.0.2, matplotlib 3.10.9, and seaborn 0.13.2.

## 3. Results

### 3.1. Characteristics of Study Population

A total of 119 participants were recruited based on the inclusion and exclusion criteria. Of these, 98 participants with complete data for all variables were included in the clustering analysis (Figure S1). The characteristics of the participants are listed in Table 1. There were 70 males and 28 females, with a mean age of 57.5 years. Forty-one participants had COPD, and 56 had a history of smoking. According to the J-CHS criteria, nine patients were frail, 49 were pre-frail, and 40 were robust. In the KCL, 24, 33, and 41 patients were frail, pre-frail, and robust, respectively. The physician diagnosed 10, 48, and 40 patients as frail, pre-frail, and robust, respectively (Tables S15–S17). Of the 98 participants, 41 had COPD, 20 had non-COPD respiratory diseases (asthma, bronchiectasis, and chronic respiratory tract infection), and 37 had no respiratory disease (Table S18).

We selected 13 variables related to physical frailty and lung function, including right handgrip strength (HS), left HS, right lower limb strength (LLS), left LLS, whole-body phase angle (WBPhA), skeletal muscle mass index (SMI), 5 m walk test (5 m WT) speed, 6 min walk test (6 min WT) speed, disease history, smoking status, percentage of predicted values of vital capacity (%VC), forced expiratory volume in one second (%FEV_1_), and forced expiratory volume in one second/ forced vital capacity (FEV_1_/FVC% or FEV_1_%). These variables were measured during the aforementioned data collection.

### 3.2. Descriptive Statistics

Comprehensive statistical summaries were generated to provide an overview of the dataset characteristics and to understand the variability within different clusters. The overall dataset and individual clusters were analyzed to determine the mean and standard deviation for each parameter. For categorical variables, the mean values corresponded to the probability of occurrence of each label, providing a clear indication of the distribution patterns within the dataset. The statistics for each parameter in the baseline measurement dataset, as well as for each of the four clusters divided using hierarchical clustering, are detailed in Table 2.

### 3.3. Spectral Co-Clustering Analysis of Parameter Correlations

**Figure 1:**
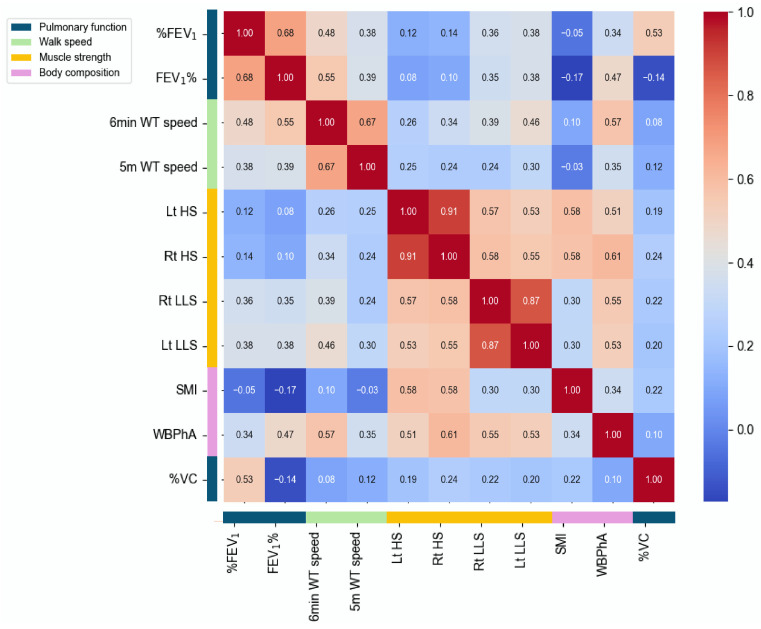
Correlation matrix of parameters from baseline measurements obtained by applying spectral co-clustering. The columns and rows are reordered to group combinations with high correlation coefficients along the diagonal. The numbers within the cells represent the correlation coefficients between parameters.

Figure 1 presents a heatmap based on the correlation coefficients among the parameters from baseline data collection, with the application of Spectral Co-clustering to identify groups with internal correlations. The analysis revealed that parameters such as %FEV_1_, FEV_1_%, 6 min WT speed, and 5 m WT speed were tightly linked, indicating a cluster of respiratory and performance measures that were closely associated. Additionally, another group emerged, consisting of HS (both right and left), LLS (both right and left), SMI, and WBPhA, all of which share rather high correlations and represent muscle strength and body composition metrics. Conversely, %VC, while moderately correlated with %FEV_1_, stood apart from these groups, highlighting its rare distinct role in the dataset.

### 3.4. Hierarchical Clustering

The number of clusters is a critical parameter in hierarchical clustering analyses. To determine the optimal cluster number, the ARI [27] was calculated for the baseline dataset with cluster numbers 3, 4, and 5 against three known labels: Japanese version of the CHS (J-CHS), KCL and Physician Classification (Table S19). ARI measures the similarity between the clustering output and predefined labels by stratifying the data. The ARI for “J-CHS” peaked at 0.16 with three clusters, followed by scores of 0.14 and 0.10 for four and five clusters, respectively. Similar trends were observed for “KCL,” where the ARI scores were 0.18, 0.16, and 0.11 with three, four, and five clusters, respectively. The “Physician Classification” also showed an optimal ARI of 0.27 at three clusters, with slight reductions to 0.22 and 0.18 for four and five clusters. The ARI was consistently highest with three clusters, indicating that the result of hierarchical clustering with three clusters showed a closer match with the known categorizations.

The internal validity indices did not identify a single optimal number of clusters. The silhouette and Calinski–Harabasz indices were highest at k = 2 and decreased monotonically (silhouette: 0.26, 0.21, and 0.19 for k = 2, 3, and 4), the gap statistic increased monotonically across k = 2 to 7 and did not exceed k = 3 under the 1-SE criterion (Figure S3; Table S7), and the cophenetic correlation was moderate (r = 0.59). With generally low bootstrap stability (mean Jaccard < 0.6 for all clusters at both k = 3 and k = 4; Figure S4), these results indicate a continuous frailty spectrum rather than well-separated discrete clusters, for which a single optimal cluster number is not well defined.

Consensus clustering then assessed whether reproducible subgroups could nonetheless be identified. The consensus matrices showed a clear block structure at both k = 3 and k = 4 (Figure S5), with similar PAC values (0.34 and 0.39), indicating that the four-cluster solution preserves stable cores comparable to those of the three-cluster solution. Consensus membership was highest and most homogeneous for Cluster 3 (mean 0.87, SD 0.03), the most severely impaired group, whereas the less impaired clusters had more diffuse boundaries (mean membership 0.65–0.71; Table S8). We acknowledge that the statistical evidence does not uniquely favor a four-cluster solution; these results are consistent with a continuous frailty spectrum rather than discrete, well-separated clusters. The four-cluster model was selected on clinical grounds: the subdivision of the most impaired group into two distinct subgroups (Clusters 2 and 3) captured a clinically meaningful distinction between moderate (COPD-predominant, preserved grip strength) and severe impairment (lowest walking speed, lowest WBPhA, highest COPD prevalence). We therefore emphasize that the four-cluster classification is exploratory and hypothesis-generating and that validation in larger cohorts is required before it can be considered an optimized or definitive solution. Notably, Clusters 0 and 1 in the three-cluster arrangement remained unchanged in the four-cluster arrangement, whereas the third cluster, when grouped into three clusters, was subdivided into Clusters 2 and 3 in the four-cluster configuration.

The distribution of the initial measurement data across the four clusters was as follows: Cluster 0, Cluster 1, Cluster 2, and Cluster 3 contained 26, 32, 21, and 19 members, respectively. For the follow-up data, the clusters had two, eight, 19, and 10 members, respectively.

When clustering was repeated using only the eleven continuous variables, the four-cluster solution showed moderate agreement with the original (ARI = 0.55), and the four-stage robust-to-frail gradient was fully preserved, with a composite severity score increasing monotonically across clusters (Table S9). The two less impaired clusters (Cluster 0, “High Function”; Cluster 1, “Low Muscle”) were almost entirely reproduced (25 of 26 and 24 of 32 members retained; Figure S6). Reassignments were concentrated in the more impaired Clusters 2 and 3 and largely confined to subjects near the cluster boundaries (Figure S7), which reorganized into a COPD-predominant airflow-obstruction cluster (mean FEV_1_%: 63.8; 21 of 31 with COPD) and a cluster with the most severely reduced pulmonary function, exercise tolerance, and lower limb strength (mean FEV_1_%: 59.3; 6 min walking speed 52.6 m/min; 13 of 16 with COPD). The multi-stage frailty structure thus did not depend on the categorical coding, whereas disease and smoking information specifically aided the subdivision of the more frail, COPD-predominant subjects.

### 3.5. Principal Component Analysis

**Figure 2:**
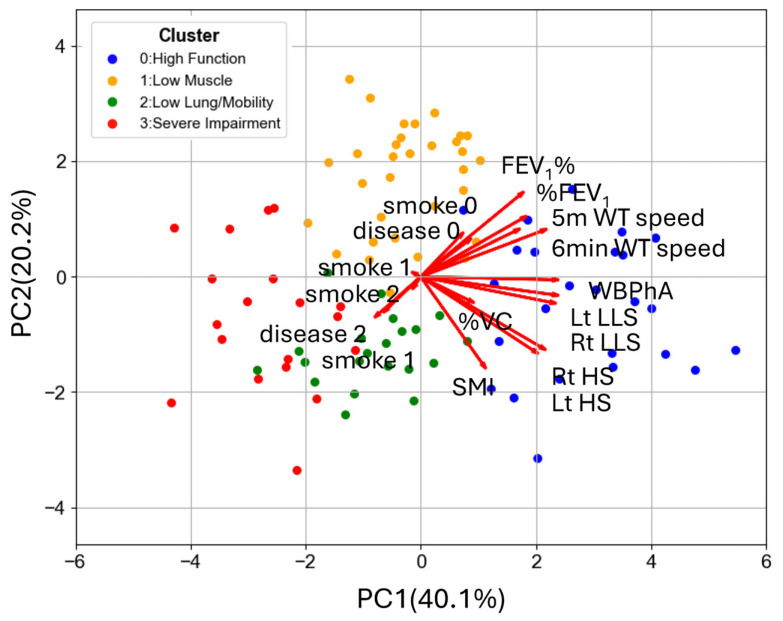
PCA scatter plot of the baseline data projected onto PC1 (40.1% variance explained) and PC2 (20.2%). Points are color-coded by cluster (0: blue, 1: orange, 2: green, 3: red). Vectors indicate the loadings of original parameters.

Principal component (PC) analysis (PCA) was applied to the data collected during the baseline measurements. Figure 2 shows the projection of the data onto the plane spanned by the two principal components with the highest variance. Each cluster was visually distinguished by a different color in the scatter plot. Cluster 0 was primarily located in the bottom right, Cluster 1 in the upper area, Cluster 2 in the bottom center, and Cluster 3 in the lower left, indicating clear separation and consistency in features within each cluster.

Overlaid on the PCA plot is a biplot that visualizes the original variables based on their loadings on the two PCs. FEV_1_%, %FEV_1_, 5 m speed, and 6 min speed cluster together diagonally from the lower left to the upper right, while WBPhA, LLS (left and right), and HS (left and right) trend in the opposite direction, from the lower right to the upper left. This result aligns well with the grouping identified through the correlation analysis, confirming the consistency between the PCA and correlation coefficients (Figure 1).

**Figure 3:**
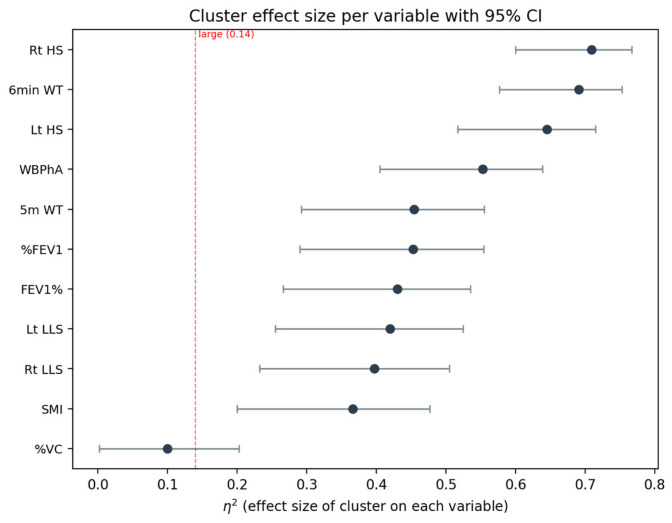
Effect sizes (η^2^) for between-cluster differences in each physical function variable. Variables are ordered by descending η^2^, estimated from one-way ANOVA across the four clusters (*n* = 98). Horizontal bars show point estimates with 95% confidence intervals (whiskers). The vertical dashed line marks the conventional threshold for a large effect (η^2^ = 0.14). Handgrip strength (right, η^2^ = 0.71; left, 0.65), 6 min WT (0.69), and WBPhA (0.55) show the largest between-cluster separation, whereas %VC (0.10, with the CI crossing the threshold) contributes least. Abbreviations: Rt, right; Lt, left; HS, handgrip strength; LLS, Lower Limb Strength; 6 min WT, 6 min walk test; 5 m WT, 5 m walk test; WBPhA, whole-body phase angle; SMI, skeletal muscle mass index; %VC, percentages of predicted vital capacity; %FEV_1_, percentage of predicted forced expiratory volume in one second; FEV_1_%, FEV_1_/FVC%.

Clusters differed significantly on all eleven variables (ANOVA, all BH-corrected *p* < 0.02). Effect sizes confirmed that the separation was driven primarily by muscle strength and mobility: grip strength (right η^2^ = 0.71, left η^2^ = 0.65), 6 min walking speed (η^2^ = 0.69), whole-body phase angle (η^2^ = 0.55), 5 m walking speed (η^2^ = 0.45), and forced expiratory function (%FEV_1_ η^2^ = 0.45; FEV_1_% η^2^ = 0.43), all exceeding the conventional threshold for a large effect (η^2^ ≥ 0.14; Figure 3; Table S6). Vital capacity (%VC) showed the weakest, though still significant, separation (η^2^ = 0.10, *p* = 0.019). 

The cluster labels reflect the domains in which each group showed its most pronounced, statistically supported deviations. The High Function cluster (Cluster 0) had the highest values across all domains (grip strength, 46.0 ± 6.8 kg; 6 min walking speed, 79.7 ± 7.3 m/min). The Low Muscle cluster (Cluster 1) was distinguished by markedly reduced grip and lower limb strength (grip strength 25.1 ± 5.0 kg) with preserved pulmonary function (%FEV_1_ 95.9; FEV_1_% 80.6). The Low Lung/Mobility cluster (Cluster 2) showed an obstructive pulmonary pattern (FEV_1_% 61.3; %FEV_1_ 70.2) and reduced walking speed (5 m walking speed 68.8 m/min) with relatively preserved grip strength (34.9 kg), consistent with its predominance of COPD (16 of 21). The Severe Impairment cluster (Cluster 3) had the lowest values across nearly all domains (6 min walking speed 46.2 m/min; whole-body phase angle 4.4°; lower limb strength 21.5 kgf), together with obstructive pulmonary impairment (FEV_1_% 58.8; COPD 17 of 19). That said, these labels should be regarded as data-driven descriptive designations rather than definitive classifications. Importantly, the pulmonary distinction between Clusters 2 and 3 was driven by forced expiratory measures rather than vital capacity, in line with the negligible effect size of %VC. Of the 66 pairwise comparisons, 44 were significant by Tukey’s HSD, and the results were essentially unchanged under the variance-robust Games–Howell test (46 of 66 significant; Table S5), indicating that the cluster distinctions were not artifacts of unequal within-cluster variances.

Age and sex did not independently account for cluster membership. As expected for a cohort spanning healthy young adults to frail older individuals, age (ANOVA F = 23.47, *p* < 0.001) and sex (χ^2^ = 57.51, *p* < 0.001) differed significantly across clusters, with the High Function cluster comprising the youngest participants (mean age, 38.4 ± 19.5 years) and the Severe Impairment cluster the oldest (78.3 ± 6.9 years) (Table S10). Age correlated moderately with PC1, the physical function axis (Pearson r = −0.68, *p* < 0.001; R^2^ = 0.46), consistent with age-related functional decline (Figure S9).

After adjusting for age and sex, the physical function axis remained the dominant predictor of cluster membership in ordinal logistic regression (PC1: OR = 0.005 per 1 SD, *p* < 0.001), whereas neither age (*p* = 0.78) nor sex (*p* = 0.24) retained an independent association (Table S11). This was not attributable to collinearity, as all VIFs were low (PC1 = 2.18, PC2 = 2.90, age = 2.52, sex = 2.71; Table S12). Model comparison was informative (Table S13): age and sex alone explained little variance (pseudo-R^2^ = 0.195) versus the physical function components alone (pseudo-R^2^ = 0.583); adding age and sex to the function model yielded negligible improvement (ΔR^2^ = +0.005), and the function-only model had the lowest AIC (121.6 vs 124.2 for the full model). Finally, all eleven physical function variables retained significant cluster effects after adjustment for age and sex and BH correction (all adjusted *p* ≤ 0.0055; Table S14). The distribution of age and sex across the principal component plane is shown in Figure S8.

### 3.6. Cluster Composition Analysis

**Figure 4:**
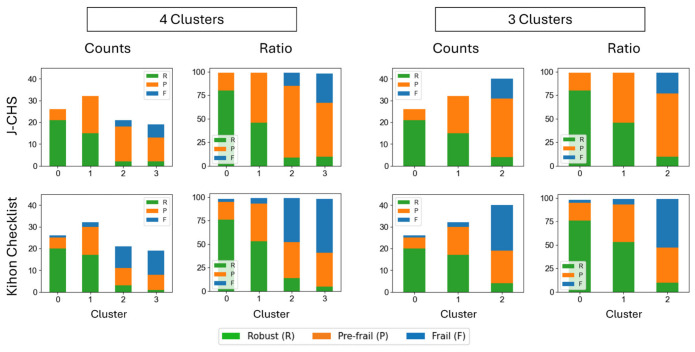
The compositions of three indices (J-CHS and KCL) within each cluster for the baseline measurement. The exact numbers of subjects are displayed as counts and ratios. (**Left panels**): 4-cluster; (**right panels**): 3-cluster configuration. For all three indices, R represents robust, P represents pre-frail, and F represents Frail. CHS, Cardiovascular Health Study.

Figure 4 illustrates the distribution and proportions of cluster memberships based on J-CHS and KCL categories using hierarchical clustering for the data collected at the baseline measurements. The graphs depict the classification results across two scenarios: four clusters (left two columns) and three clusters (right two columns) for the respective J-CHS and KCL categories. In these bar graphs, Label R represents “Robust,” Label P represents “Pre-frail,” and Label F denotes “Frail” individuals.

In the three-cluster configuration, Cluster 2 predominantly contained subjects classified as “Pre-Frail” and “Frail” in both the J-CHS and KCL categories, indicating that this cluster consisted of frail subjects. Meanwhile, in the four-cluster configuration, the single Cluster 2 in the three-cluster scenario was subdivided into Clusters 2 and 3 (Table 2 and Table S20). Again, Cluster 2 mainly comprised “Pre-Frail” and “Frail” subjects; however, Cluster 3 showed a higher proportion of ‘Frail’ subjects, characterizing it as a cluster with more advanced frailty stages. This trend was also consistent with the physician classifications, in which Cluster 2 was predominantly composed of ‘Pre-Frail’ subjects, while Cluster 3 contained the majority of ‘Frail’ subjects (Figure S10).

In Cluster 0, although some “Pre-frail” and a few “Frail” subjects were included, a substantial majority of individuals were classified as “Robust,” indicating that this cluster predominantly consisted of individuals without significant signs of frailty. In contrast, Cluster 1 included a similar proportion of “Robust” individuals as Cluster 0 but had a noticeably lower percentage of individuals. This shift suggests that, while Cluster 1 also comprised generally robust subjects, it represented a transitional stage where signs of frailty were more apparent than in Cluster 0.

These observations suggest a gradation in the progression of frailty across the clusters, with Clusters 0, 1, 2, and 3 successively representing a spectrum from robust to more pronounced frailty conditions.

**Figure 5:**
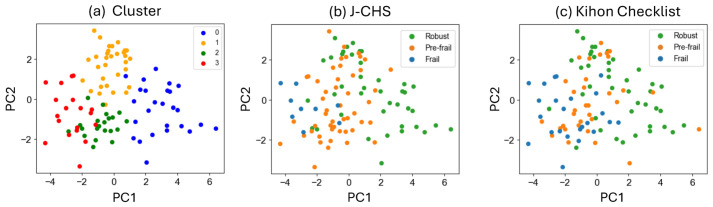
PCA scatter plots (PC1 vs. PC2) of baseline data, color-coded by (**a**) four clusters, (**b**) J-CHS, and (**c**) KCL, showing the correspondence between clusters and index levels. Cluster labels: 0 = High Function, 1 = Low Muscle, 2 = Low Lung/Mobility, 3 = Severe Impairment. CHS, Cardiovascular Health Study.

Figure 5 shows the projection of subject data onto a two-dimensional plane spanned by PC1 and PC2 for the baseline data measurement. The figure includes three panels, each corresponding to a different categorization method: (a) cluster number, (b) J-CHS, and (c) KCL. A consistent pattern emerged across the J-CHS, and KCL panels, where individuals with lighter frailty levels tended to appear on the right side of the distribution, while those with more severe conditions were grouped on the opposite side, specifically towards the lower-left region of the plots. A similar tendency was observed in the Physician Classification (Figure S11). This distribution illustrates a clear gradient of health conditions, where one end of the principal component spectrum was associated with healthier individuals and the opposite end with individuals facing more severe health challenges.

The alignment of these health condition levels with the four cluster positions clarifies the meaning of the clusters, as described in the previous section. Specifically, there was an observable gradation of frailty severity across the clusters, with Cluster 0 being the least frail and severity increasing through Clusters 1, 2, and 3.

These PCA plots not only validate the applied clustering logic but also visually depict the multidimensional nature of health variability within the studied population, enhancing the understanding of the relationship between different health assessment criteria and patient distribution in the principal component space.

### 3.7. Transition of Subject States from First to Second Measurement

**Figure 6:**
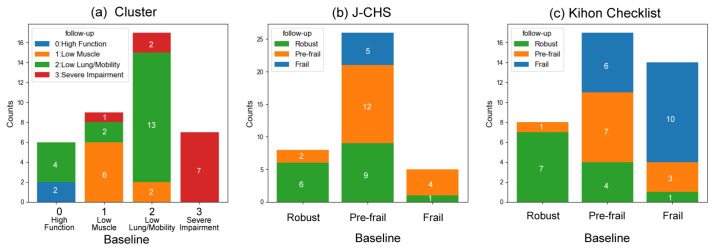
Index transitions from baseline to follow-up: (**a**) four clusters, (**b**) J-CHS, and (**c**) KCL. Horizontal axis: baseline levels; vertical axis: count. Stacked bars show the follow-up distribution (numbers = individual counts). *n* = 39. CHS, Cardiovascular Health Study.

Figure 6 illustrates the transitions of the subjects’ states between baseline and follow-up measurements based on cluster assignment and health status categories: J-CHS and KCL. The figure comprises three panels (a–c), each depicting the transitions in cluster assignment (a) and the levels of J-CHS (b), and KCL (c).

In each panel, the x-axis represents the cluster or level of frailty at the baseline measurement, while the vertical axis represents the count of individuals. The bars indicate the distribution of these subjects into clusters or levels at the follow-up measurement, quantified on the y-axis. Each bar is stacked to show the distribution of follow-up levels, with numbers indicating the detailed count of subjects in each category. For example, panel (a) focuses on cluster transitions. At baseline measurement, six subjects belonged to Cluster 0, of whom two remained in Cluster 0, whereas four moved to Cluster 2 in the follow-up measurement, indicating progression in frailty. Among the nine subjects initially in Cluster 1, six remained within the same cluster, two progressed to Cluster 2, and one advanced to Cluster 3, showing varying degrees of frailty progression. Of the 17 subjects originally in Cluster 2, two improved and moved to Cluster 1, suggesting mitigation of frailty symptoms, most remained, and two progressed to Cluster 3. All seven subjects in Cluster 3 remained in the same cluster during follow-up.

Cluster 3 showed no change over time, which was consistent with the Physician Classification (Figure 6a and Figure S12), whereas the CHS and KCL frailty classifications showed changes over time (Figure 6b,c).

**Figure 7:**
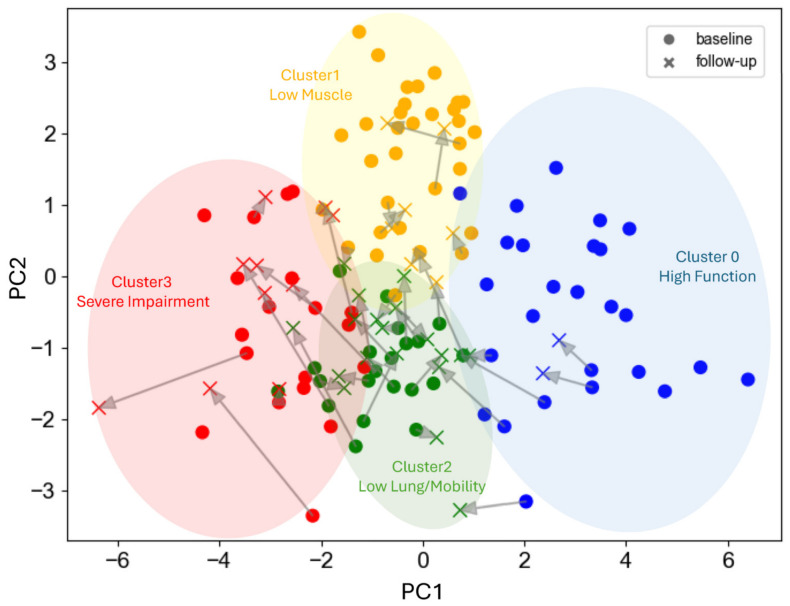
Cluster transitions from baseline to follow-up on the PC1-PC2 plane. Circles (○): baseline (*n* = 98); crosses (×): follow-up (*n* = 39). Arrows indicate the trajectories of individuals measured at both timepoints; colors denote clusters. PC, principal component.

The scatter plot in Figure 7 illustrates the transitions of the subjects between the two measurements on a PCA-reduced two-dimensional plane.

This approach effectively captures the dynamic changes in subjects’ cluster positions, offering a vivid representation of how individual conditions evolved or remained stable over time. For the 39 subjects who underwent follow-up measurement (Table S21), the arrows indicate the direction of change in their cluster positioning. Most subjects moved to the upper left, and very few moved to the right. This visualization not only facilitates understanding of the progression or regression of health conditions, as represented by the cluster transitions, but also illustrates the effectiveness of the clustering methodology in capturing clinically meaningful changes in patient status. However, these longitudinal observations are based on only 39 of the 98 participants who could be re-measured, and some follow-up clusters contained very few subjects (two to 19); the corresponding results should therefore be regarded as exploratory.

The arrangement of subjects on the PCA-reduced two-dimensional plane not only allows for the evaluation of an individual’s current frailty based on their position but may also illustrate possible directions of change over time. It should be emphasized that these trajectories are descriptive visualizations derived from a two-dimensional PCA projection and from a small follow-up sample (*n* = 39); they do not constitute a validated predictive model. Quantitative longitudinal modeling in larger cohorts would be required before the approach could be used to predict individual frailty progression or to guide the timing of interventions. With these caveats, trajectory visualization may help generate hypotheses about how a patient’s condition could evolve, which could inform future studies of intervention strategies.

## 4. Discussion

In this study, using unsupervised machine learning, we reclassified frailty—previously captured in three categories—into four more granular groups spanning a spectrum from robust to pronounced frailty, in alignment with existing frailty indices. The component loading plot (biplot) showed variables aggregating in two directions: one related to cardiopulmonary function (exercise tolerance) and the other related to physical elements, which could be therapeutic targets based on the location of the subject’s data points.

Many machine-learning studies that used gait or physical activity data to analyze frailty have been reported [28]. These data are collected from various motion sensors, such as Kinect movement sensors [29], home sensors [30], and the most popular wearable sensors [31,32,33,34,35]. Razjoujan et al. used pendant sensors to detect cognitive frailty, using criteria derived from the CHS and the Mini-Mental State Examination. They showed that everyday activity parameters (e.g., cumulative postures, activity behavior, and locomotion) derived from a single chest-worn sensor could distinguish older adults with cognitive frailty [33]. As Clegg et al. presented the electronic Frailty Index [36], in addition to physical and motor function parameters, Electronic Health Records are also frequently analyzed [37,38]. These records often include subjective or semi-objective data, making it difficult to judge based on objective data. Most of these studies employed supervised machine learning to detect or predict the frailty status. Only a few studies have attempted detailed classification of frailty using unsupervised machine learning, as in this study. Abbas et al. performed PCA using acceleration-based gait data obtained from wearable sensors. Although the data were divided into three clusters (representing frail, prefrail, and robust CHS criteria), no further classification was made, and there was no mention of the continuity of classification [34]. Nejatifar Z et al. assessed the palliative care needs of patients with COPD using a super learning approach based on clinical data—such as low BMI, fatigue status, physical activity level, slow walking speed, and FEV_1_—without the use of sensors [39]. Except for Razjoujan et al.’s attempt to detect cognitive frailty [33], most studies aimed to measure frailty status or predict mortality, hospitalization, or nursing home admission; none addressed the type of appropriate intervention. Compared with these previous machine-learning studies, our approach differs methodologically in that it applies unsupervised clustering to directly measured clinical and physiological variables rather than to sensor-derived gait signals, and it emphasizes a continuous, multidimensional representation of frailty; conversely, it shares with them the common limitations of a modest sample size and the absence of external validation.

To the best of our knowledge, this is the first study to perform unsupervised learning using actual measurements of lung function and multifaceted physical function in respiratory diseases. The strength of this study is that data scientists and clinical professionals collaborated closely to analyze these factors, including objectively measured clinical variables, such as lung function, 5 m WT speed, 6 min WT speed, handgrip strength, and body composition (SMI and WBPhA). Because it is based on objective measurements, this model is highly reproducible and is not subject to the biases inherent in subjective questionnaires. Most studies that use gait-related measurement data have adopted supervised learning to detect frailty automatically using various sensors, with frailty categories defined by the CHS used as label data. We attempted to further classify frailty using actual measurement data based on multidimensional physical function to determine the appropriate timing and parameters for interventions in frailty progression.

In the spectral co-clustering analysis (Figure 1), %FEV_1_, FEV_1_%, 6 min WT speed, and 5 m WT speed were closely linked, indicating a group derived from lung function and exercise tolerance. Moreover, there were associations between HS (right and left), LLS (right and left), SMI, and WBPhA, which represent physical elements. This relationship is also reflected in the two directions of the vectors in the biplot, as shown in Figure 2. Therefore, depending on the subject’s position on the plane spanned by the 2-PC plane, it may be possible in future prospective studies to explore individualized therapeutic strategies targeting specific cardiopulmonary or physical functions, though this warrants validation through intervention research.

Hierarchical clustering and PCA enabled us to visualize the transition from robust to frail and track the changes in this study. Participants were divided into four clusters, each representing a position along the robust-to-frail spectrum based on the characteristics of its members (Table 2, Figure 4). By examining cluster membership, frailty could be assessed in a more granular, discrete manner for each subject. The directions of the parameters in the biplot (Figure 2) clearly show the differences between the four clusters; for example, the differences between Clusters 2 and 3 were grip strength and SMI, and the differences between Clusters 0 and 2 were %FEV_1_, FEV_1_%, 6 min WT speed, and 5 m WT speed. Cluster 1 exhibited traits commonly associated with females, such as lower grip strength, a lack of smoking history, and frequent asthma, with 78% of its members being female. This cluster also suggests a need for improved muscle strength (Table 2, Figure 2). Furthermore, the relationship between the positions of the data points on the 2-PC plane and the PC loadings allowed for the evaluation of differences not only among clusters but also within clusters. This enabled a more detailed understanding of each subject’s condition and may potentially inform the selection of intervention targets in future prospective studies. The change in the data points in Figure 7 suggests that the direction of the vectors shown in the biplot could provide a basis for tailoring treatments targeting cardiopulmonary and physical functions.

The quantitative characterization placed the previously descriptive cluster labels on a firmer statistical footing, with large effect sizes (η^2^ up to 0.71) and 95% confidence intervals for muscle strength, mobility, body composition, and forced expiratory function (Figure 3). Notably, the pulmonary component of the impaired clusters was attributable to obstructive measures (FEV_1_%, %FEV_1_) rather than vital capacity, consistent with the high prevalence of COPD in Clusters 2 and 3.

Under the conventional classification, 9 participants were classified as frail according to the J-CHS, 24 according to the KCL, and 10 according to the Physician Classification. In contrast, in the new model, 19 people were classified into Cluster 3, which was estimated to represent the most severe frailty. There was, however, some overlap between the ‘frail’ category and Cluster 3 in each classification (Figure 5). When follow-up measurements were analyzed, many participants’ results were plotted near their baseline positions, indicating a high level of reproducibility in the results (Figure 7). Most data points shifted slightly toward the upper left over time, indicating age-related declines in physical parameters, such as HS (both right and left), LLS (both right and left), and SMI, with age (Figure 2 and Figure 7). In this study, only a few significant changes in symptoms were observed over a mean interval of 636 days, suggesting that noticeable alterations may require a longer time to manifest. These results suggest that frailty in patients with respiratory diseases leads to a decline in physical function before notable deterioration of respiratory function and endurance, especially within a shorter time frame.

Figure 6 shows the changes over time in participants according to cluster and the J-CHS and KCL frailty criteria. Cluster 3 showed no transition in subject status, indicating that it represents the most severe frailty stage. This result was consistent with the frail category as diagnosed by the Physician Classification (Figure S12). In contrast, frail subjects in J-CHS and KCL improved to pre-frail or robust. Previous studies have suggested that the rate of improvement in frailty status is generally very low [40,41], and the results of this transition study support the validity of frailty assessment through clustering and the Physician Classification. Furthermore, the concordance between cluster-based frailty assessment and the Physician Classification suggests that this approach may capture aspects of frailty not detected by questionnaires or simple tests alone. Additionally, the reproducibility of these cluster-based assessments may help inform the timing of interventions. In essence, cluster classification may have the potential to complement certain aspects of physician-driven assessments, potentially supporting structured medical support through future comparisons with a greater number of physician classifications.

Satake et al. reported that the KCL score was closely correlated with the number of frailty phenotypes defined by the CHS; this finding aligns with the results of this study [22]. The composition of the J-CHS criteria in the four clusters was similar to that of the KCL criteria, which included psychological and social frailty factors (Figure 4). Interestingly, the ARI score for this 4-cluster model against the KCL (0.16) was slightly higher than that of the J-CHS (0.14). Although the four new clusters were developed based on physical function, the results showed no inconsistency with conventional questionnaire-based frailty assessments, which also included social and psychological aspects. The consistency of these results with established assessments is important, as it has the potential to provide more granular and continuous patient assessment without contradicting previous findings, which could, in future studies, serve as a foundation for exploring more personalized treatment approaches.

The sensitivity analysis excluding categorical variables (ARI = 0.55 with the original solution) indicated that disease status and smoking history contributed substantially to cluster assignment, particularly to the subdivision of the more impaired clusters. This raises an important interpretive question: does the model primarily capture frailty status, or does it reflect respiratory disease severity? The data suggested that both were operating simultaneously. When only continuous physical function variables were included, the four-stage robust-to-frail gradient was preserved, indicating that the frailty structure did not depend entirely on disease categorization. However, disease and smoking information specifically refined the separation of COPD-predominant frail subjects (Clusters 2 and 3). The present model should therefore be understood as capturing a combined phenotype of physical frailty and respiratory impairment, which may, in fact, be clinically appropriate for a respiratory disease cohort but may limit its generalizability to frailty assessment in non-respiratory populations.

Resampling analyses indicated that, although individual cluster boundaries were sensitive to perturbation, the cluster cores were reproducible, with the most severely impaired group (Cluster 3) forming the most stable core (consensus membership: 0.87). This is clinically reassuring, as patients with the most advanced frailty—those for whom accurate recognition matters most—were identified most reliably. The continuous nature of the data explains why conventional internal indices did not converge on a single optimal cluster number and why centroid- and density-based alternatives were unstable; the deterministic, reproducible four-stratum hierarchical solution is better suited to an eventual clinical tool than a purely statistical determination of cluster number.

The relatively low bootstrap stability (mean Jaccard < 0.6 for all clusters) reflects the continuous nature of the frailty spectrum in this cohort rather than the presence of distinct natural groupings. This instability implies that individual cluster boundaries—particularly between adjacent clusters—are sensitive to perturbation and should not be interpreted as fixed thresholds. Clinically, this means that individual patients near cluster boundaries may be classified differently across repeated measurements or in different samples. For clinical implementation, a continuous frailty score derived from the principal component plane may ultimately be more appropriate than a discrete four-class label. Replication in larger, more diverse cohorts is necessary to determine whether stable, reproducible boundaries can be identified.

The cohort spanned a wide age range and included healthy young medical students in the High Function cluster, raising the possibility that the cluster structure merely reflected age and sex. Our adjusted analyses argue against this interpretation. Although age was moderately correlated with the physical function axis (r = −0.68), it carried no independent predictive information once physical function was accounted for (adjusted *p* = 0.78; incremental pseudo-R^2^ = +0.005), and low VIFs confirmed that this was not an artifact of collinearity. These findings indicate that the influence of age on cluster membership operates indirectly, mediated through measurable declines in physical function, rather than as an independent confounder. In other words, the clusters appear to capture the physiological consequences of aging as they are imprinted on objective physical function measures, rather than age per se. We nonetheless acknowledge that the inclusion of healthy young participants broadens the functional spectrum and that validation in a more age-restricted cohort would further strengthen these conclusions.

This study had some limitations. The sample size (*n* = 98) may be considered small or insufficient, and all participants were recruited from a single university hospital. The absence of external validation is a critical limitation. As this model was developed and evaluated in a single-center cohort, its performance in other populations, institutions, and healthcare systems remains uncertain. External validation in independent, multicenter cohorts is necessary before this clustering approach can be considered for clinical application. A key structural limitation of the present study is the inclusion of young medical students in the healthy control group, which results in a structural age imbalance that cannot be fully accounted for by statistical adjustment. Consequently, the robust group was much younger than the frail and pre-frail groups, as the proportion of healthy participants was higher among students than among outpatients. Therefore, more healthy older adults should be included in future studies. There were fewer females in this cohort, and male and female participants were analyzed together. Gender bias was observed in the clusters, and gender differences should also be considered in future research. The results of the clusters in this study were most similar to the experienced Physician’s Classification; however, to confirm these results, multiple Physician Classifications must also be taken into consideration in future research. As mentioned in Section 2.2, baseline and second measurement periods varied and only 39 participants were able to undergo follow-up measurements. Therefore, the follow-up analysis was restricted to these participants; as some follow-up clusters contained very few subjects, the reproducibility and transition findings should be interpreted as preliminary. This was partly because each measurement phase spanned approximately 1.5 years and only a limited number of outpatients—but no students—could be re-measured. Additionally, measuring all 13 variables in routine clinical practice would be challenging; therefore, future studies should explore replacing them with simpler measures, such as heart rate.

Taking these limitations into account, the present study was conducted as a pilot study. Future studies should include larger and more diverse sample sizes and use long-term follow-up data to confirm these transitions.

## 5. Conclusions

This frailty assessment employed unsupervised machine learning with multidimensional physical frailty parameters in respiratory diseases, offering a novel and objective model with four detailed classifications that align with existing frailty indices. By characterizing the clusters along two functional axes (cardiopulmonary function and physical function), potential therapeutic targets along each axis could be identified. As an exploratory, hypothesis-generating pilot study, these findings require confirmation in larger, externally validated cohorts before clinical application; with such validation, the approach may, in the future, contribute to personalized frailty management.

## Figures and Tables

**Table 1:** Characteristics of the study participants.

	All (*n* = 98)
Age (years)	57.5 ± 22.3
Gender (male/female)	70/28
BMI	22.5 ± 3.7
Disease [COPD/Non-COPD/Healthy controls]	41/20/37
Smoking status [Cu/Ex/Non]	11/45/42

BMI, body mass index; COPD, chronic obstructive pulmonary disease. Data are presented as mean ± standard deviation.

**Table 2:** Summary of all parameters measured at the baseline data collection.

	Class All (*n* = 98)	Class 0 (*n* = 26)	Class 1 (*n* = 32)	Class 2 (*n* = 21)	Class 3 (*n* = 19)
	Mean ± STD	Mean ± STD	Mean ± STD	Mean ± STD	Mean ± STD
%VC (%)	98.5 ± 15.8	105.7 ± 14.3	97.3 ± 13.0	91.5 ± 16.1	98.6 ± 18.5
%FEV_1_ (%)	87.3 ± 19.5	100.6 ± 11.4	95.9 ± 12.1	70.2 ± 17.4	73.6 ± 18.6
FEV_1_% (%)	72.5 ± 15.8	81.7 ± 12.5	80.6 ± 10.5	61.3 ± 9.1	58.8 ± 16.4
5 m WT speed (m/min)	75.5 ± 16.5	85.8 ± 9.2	82.8 ± 13.4	68.8 ± 14.6	56.8 ± 11.8
6 min WT speed (m/min)	68.7 ± 14.6	79.7 ± 7.3	75.0 ± 7.2	65.9 ± 7.8	46.2 ± 11.0
Rt HS (kg)	33.0 ± 10.3	46.0 ± 6.8	25.1 ± 5.0	34.9 ± 4.8	26.6 ± 5.6
Lt HS (kg)	31.2 ± 9.4	42.1 ± 7.5	23.7 ± 4.5	34.0 ± 5.9	26.0 ± 4.2
Rt LLS (kg)	33.5 ± 17.7	51.3 ± 20.8	28.5 ± 10.5	30.1 ± 11.0	21.5 ± 9.3
Lt LLS (kg)	31.7 ± 17.2	49.3 ± 20.6	28.1 ± 9.1	26.6 ± 9.6	19.1 ± 9.4
WBPhA (°)	5.5 ± 1.1	6.8 ± 1.0	5.3 ± 0.7	5.2 ± 0.6	4.4 ± 0.7
SMI (kg/m^2^)	6.8 ± 1.0	7.5 ± 0.8	6.2 ± 0.7	7.4 ± 0.9	6.5 ± 0.9
smoke 0 (Non-smoker)	0.4 ± 0.5	0.7 ± 0.5	0.8 ± 0.4	0.0 ± 0.2	0.0 ± 0.0
smoke 1 (Ex-smoker)	0.5 ± 0.5	0.2 ± 0.4	0.2 ± 0.4	0.9 ± 0.4	0.8 ± 0.4
smoke 2 (Current smoker)	0.1 ± 0.3	0.1 ± 0.3	0.1 ± 0.2	0.1 ± 0.3	0.2 ± 0.4
disease 0 (Healthy controls)	0.4 ± 0.5	0.7 ± 0.5	0.6 ± 0.5	0.0 ± 0.0	0.0 ± 0.0
disease 1 (Non-COPD)	0.2 ± 0.4	0.1 ± 0.3	0.3 ± 0.5	0.2 ± 0.4	0.1 ± 0.3
disease 2 (COPD)	0.4 ± 0.5	0.2 ± 0.4	0.1 ± 0.3	0.8 ± 0.4	0.9 ± 0.3

Summary of the mean and standard deviation of all parameters measured at baseline data collection in the study, including smoking status and disease history, which are represented as dummy variables using one-hot encoding. The leftmost column shows the data for the entire sample, while the subsequent columns (from left to right) present the mean and standard deviation for each cluster (Cluster 0, Cluster 1, Cluster 2, and Cluster 3). Smoking status is originally a categorical variable with values 0, 1, and 2, where 0 (smoke 0 = 1) indicates non-smoker, 1 (smoke 1 = 1) indicates ex-smoker, and 2 (smoke 2 = 1) indicates current smoker. Due to the dummy variable transformation, the mean values in the table correspond to the proportion of each category within the entire data or clusters. Similarly, disease history is a categorical variable with values 0, 1, and 2, where 0 (disease 0 = 1) indicates healthy controls, 1 (disease 1 = 1) indicates non-COPD and 2 (disease 2 = 1) indicates COPD (including ACO). %VC, percentages of predicted vital capacity; FVC, forced vital capacity; %FEV_1_, percentages of predicted forced expiratory volume in one second; FEV_1_%, FEV_1_/FVC%; 6 min WT, 6 min walk test; 5 m WT, 5 m walk test; Rt, right; Lt, left; HS, handgrip strength; LLS, Lower Limb Strength; WBPhA, whole-body phase angle; SMI, skeletal muscle mass index; STD, standard deviation.

## Data Availability

The original data presented in this study are not publicly available due to IRB restrictions regarding participant privacy. However, data are available from the corresponding author upon reasonable request.

## Supplementary Materials

The following supporting information can be downloaded at: https://www.mdpi.com/article/10.3390/jcm15135145/s1, Figure S1: Flow chart of the included participants; Figure S2: Reproducibility of k-means clustering across random initializations (k = 4, 50 random seeds); Figure S3: Internal validation metrics as a function of the number of clusters (k = 2–7), based on Ward linkage applied to the 17 standardized variables (*n* = 98); Figure S4: Bootstrap cluster stability assessed by the Jaccard coefficient (B = 500 resamples) for the three-cluster (left) and four-cluster (right) Ward solutions; Figure S5: Consensus matrices from consensus clustering (Ward linkage, 500 subsamples at 80% subsampling) for k = 3 (left panel) and k = 4 (right panel), with subjects ordered by consensus; Figure S6: Cross-tabulation (heatmap) of cluster assignments from the original 17-variable solution (rows, clusters 0–3) versus the solution obtained after removing the six disease/smoking dummy variables (columns, clusters 1–4); Figure S7: Principal-component plane (PC1 vs PC2) showing subjects who were reassigned to a different cluster after removal of the disease/smoking dummy variables (*n* = 24, circled in black); Figure S8: Principal-component plane (PC1 vs PC2) coloured by (a) cluster, (b) age, and (c) sex (*n* = 98); Figure S9: Scatter plot of PC1 score (physical-function axis) versus age, treating age as a supplementary variable external to the PCA (*n* = 98); Figure S10: The compositions of three indices (Physician Classification) within each cluster for the baseline measurement; Figure S11: Scatter plots of data points projected onto a plane spanned by PC1 and PC2 from PCA for baseline data measurement; Figure S12: Transitions of indices from baseline to follow-up: Physician Classification; Table S1: Comparison of clustering methods by agreement with the reference Ward solution (k = 4); Table S2: Stability of k-means clustering across 50 random initializations (k = 4) under two settings (n_init = 10, the production setting; n_init = 1); Table S3: DBSCAN eps search using k-distance quantiles (min_samples = 4) on the 17-variable standardized data; Table S4: Pairwise full; Table S5: Pairwise gameshowell; Table S6: Omnibus between-cluster differences per variable (*n* = 98); Table S7: Internal validation metrics for Ward hierarchical clustering across candidate numbers of clusters (k = 2–7; 17 variables (11 standardized + 6 one-hot), *n* = 98); Table S8: Consensus membership by cluster (k = 4); Table S9: Physical-function profile of the clusters obtained after removing the disease/smoking dummy variables (observed values; mean ± SD), ordered from robust to frail; Table S10: Age and sex distribution by cluster (*n* = 98); Table S11: Ordinal logistic regression of cluster rank on PC1, PC2, age, and sex (continuous predictors standardized; *n* = 98); Table S12: Variance inflation factors (VIF) for the predictors of the ordinal regression model (PC1, PC2, age, sex); Table S13: Ordinal-model comparison quantifying the incremental contribution of physical function versus age/sex (*n* = 98); Table S14: Between-cluster differences for each physical-function variable adjusted for age and sex (linear model, type-II ANOVA; *n* = 98); Table S15: Characteristics of the study participants (J-CHS); Table S16: Characteristics of the study participants (Kihon Check List); Table S17: Characteristics of the study participants (Physician classification); Table S18: Summary of the study participants’ diseases; Table S19: Adjusted Rand Index (ARI) for clusters 3, 4, and 5 against J-CHS, KCL and Physician Classification; Table S20: Summary of all parameters measured at baseline data collection (three clusters); Table S21: Summary of all parameters measured at follow-up data collection.

## Abbreviations

The following abbreviations are used in this manuscript:

%VC	percentages of vital capacity
FVC	forced vital capacity
FEV_1_	forced expiratory volume in one second
%FEV_1_	percentages of predicted forced expiratory volume in one second
FEV_1_%	FEV_1_/FVC %
6 min WT	6 min walk test
5 m WT	5 m walk test
Rt	right
Lt	left
HS	handgrip strength
LLS	Lower Limb Strength
WBPhA	whole-body phase angle
SMI	skeletal muscle mass index
CHS	Cardiovascular Health Study
KCL	Kihon Checklist
