# Supplementary material for: Novel Frailty Assessment Based on Multidimensional Physical Frailty Parameters Using Unsupervised Clustering in Respiratory Diseases: A Pilot Study
Source: J Clin Med. 2026 Jul 1;15(13):5145. doi: 10.3390/jcm15135145 (PMC13363542; doi:10.3390/jcm15135145)
Supplement: Supplementary file 1 [file jcm-15-05145-s001.zip › Supplementary Material_final3.pdf]

Supplementary Materials

Supplementary Figure

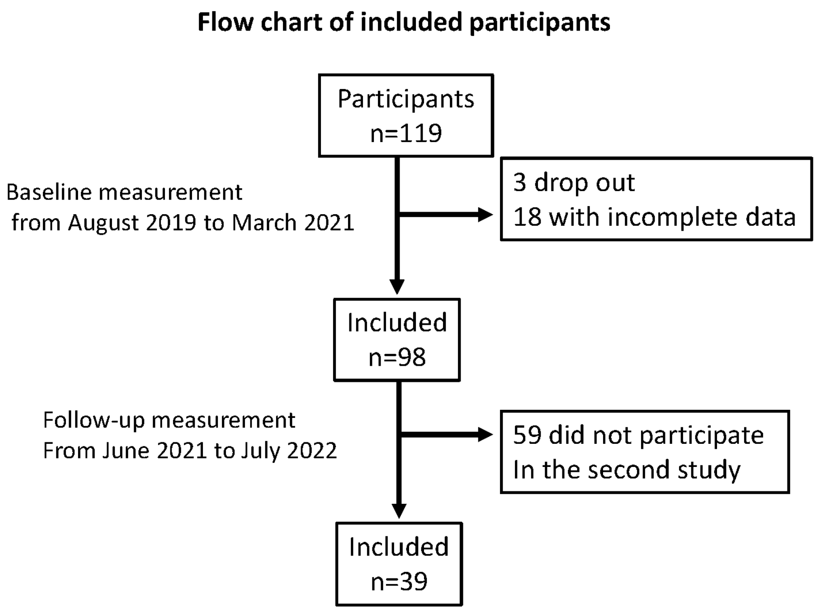

Figure S1. Flow chart of the included participants.

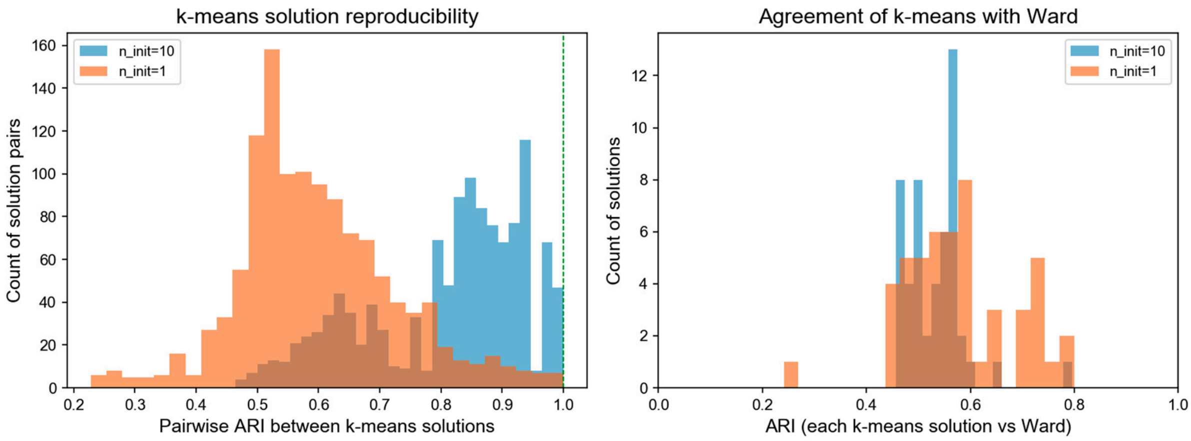

Figure S2. Reproducibility of k-means clustering across random initializations (k = 4, 50 random seeds). Left: distribution of pairwise adjusted Rand index (ARI) between the 50 k-means solutions for n_init = 10 (blue) and n_init = 1 (orange); the green dashed line marks ARI = 1.0 (identical partitions). Right: distribution of ARI between each k-means solution and the reference Ward solution. With n_init = 10, pairwise ARI averaged 0.81 (only 3.8% identical) and agreement with Ward averaged 0.53; with n_init = 1, pairwise ARI averaged 0.60. k-means did not converge to a single stable partition and showed only moderate agreement with Ward, supporting the choice of Ward hierarchical clustering.

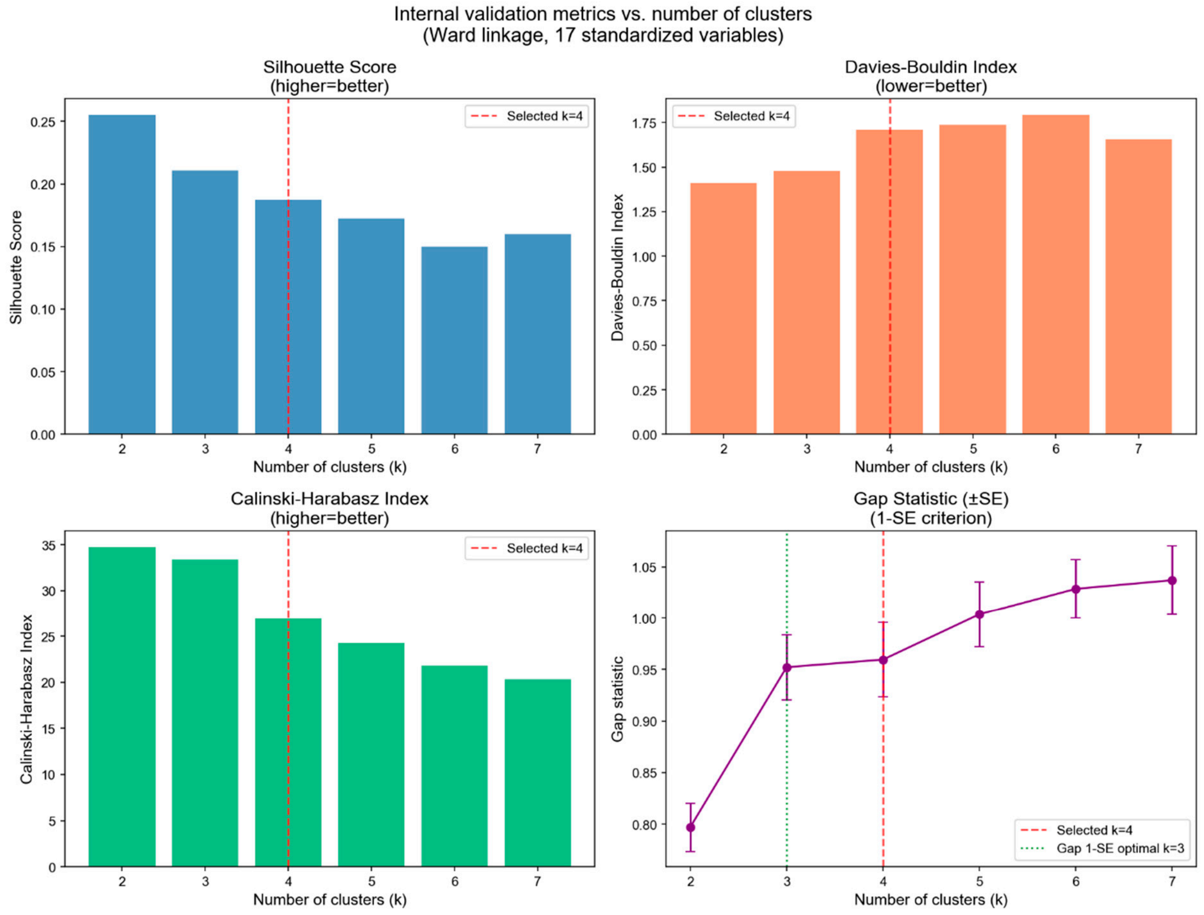

Figure S3. Internal validation metrics as a function of the number of clusters (k = 2–7), based on Ward linkage applied to the 17 standardized variables (*n* = 98). Four panels show the silhouette score (higher = better), Davies–Bouldin index (lower = better), Calinski–Harabasz index (higher = better), and the gap statistic (mean ± SE). The red dashed line indicates the selected solution (k = 4); the green dotted line in the gap-statistic panel marks the 1-SE-optimal value (k = 3). No single index identifies a unique optimum: the silhouette and Calinski–Harabasz indices decline monotonically, the gap statistic increases gradually, and the 1-SE criterion favours k = 3. The four-cluster solution was therefore selected on the basis of clinical interpretability together with the stable cluster cores demonstrated by consensus clustering (Figure S5, Table S8), rather than on any internal index alone.

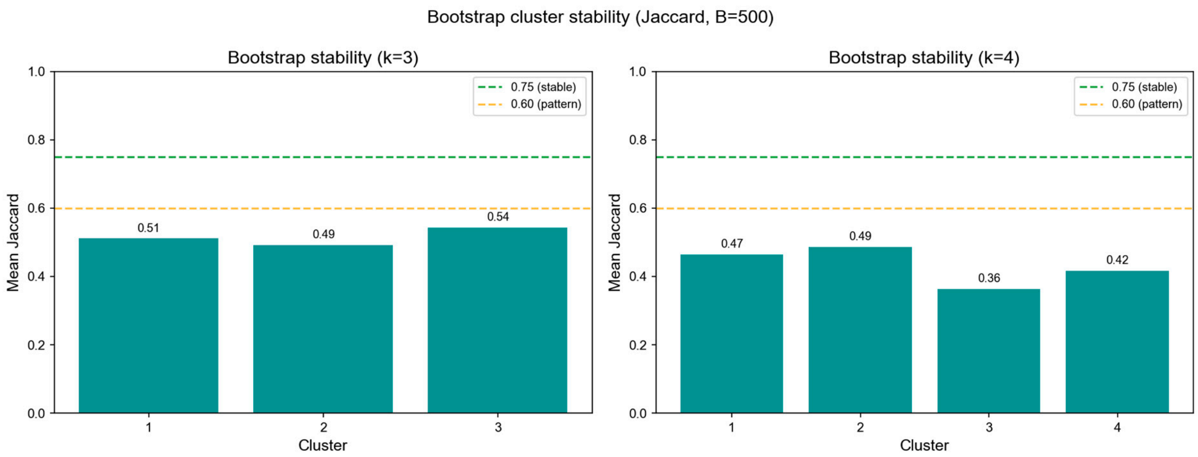

Figure S4. Bootstrap cluster stability assessed by the Jaccard coefficient (B = 500 resamples) for the three-cluster (left) and four-cluster (right) Ward solutions. Bars show the mean Jaccard index per cluster; the green dashed line (0.75) denotes the conventional threshold for a "stable" cluster and the orange dashed line (0.60) the threshold for a "pattern-bearing" cluster. All clusters fall below 0.60 under both solutions (k = 3: 0.49–0.54; k = 4: 0.36–0.49), indicating that individual cluster boundaries are not highly reproducible under resampling and are consistent with a continuous frailty gradient rather than sharply separated groups.

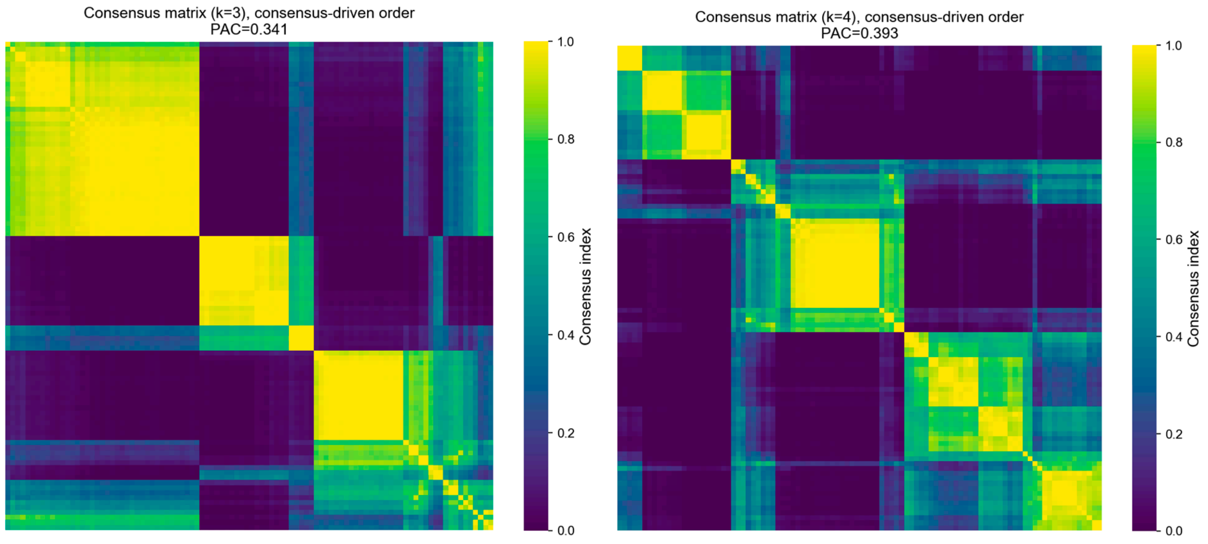

Figure S5. Consensus matrices from consensus clustering (Ward linkage, 500 subsamples at 80% subsampling) for k = 3 (left panel) and k = 4 (right panel), with subjects ordered by consensus. Color denotes the consensus index (proportion of resamples in which two subjects co-cluster; 0 = never, 1 = always). The proportion of ambiguous clustering (PAC) was 0.341 for k = 3 and 0.393 for k = 4. Well-defined diagonal blocks indicate stable cluster cores, while the off-diagonal mixing at k = 4 reflects boundary subjects whose assignment is less certain, again supporting a continuous spectrum of physical function.

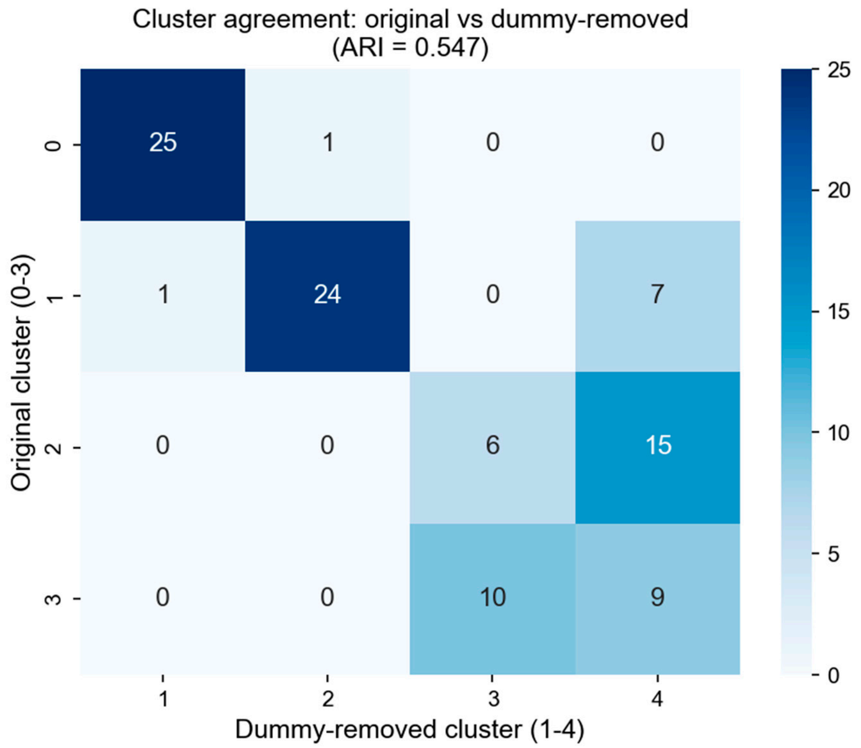

Figure S6. Cross-tabulation (heatmap) of cluster assignments from the original 17-variable solution (rows, clusters 0–3) versus the solution obtained after removing the six disease/smoking dummy variables (columns, clusters 1–4). Cell values are subject counts; color intensity is proportional to count. Overall agreement was moderate (ARI = 0.547). The High-Function (0) and Low-Muscle (1) clusters were highly preserved (25/26 and 24/32 retained), whereas the two lower-function clusters (2 and 3) exchanged members, indicating that the dummy variables mainly refine the boundary between the Low Lung/Mobility and Severe-Impairment groups.

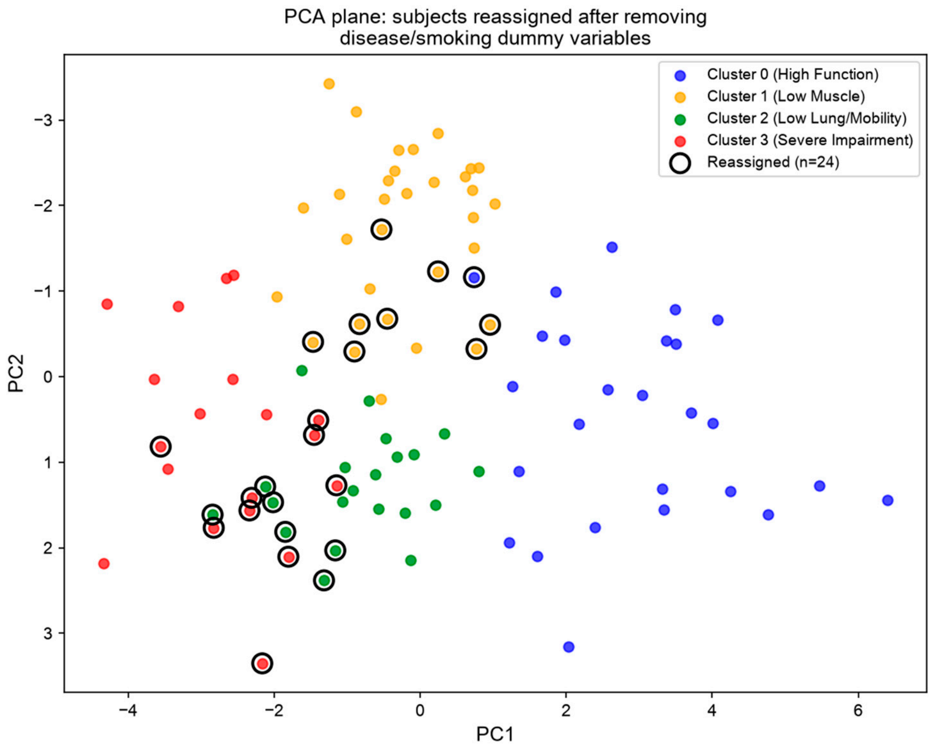

Figure S7. Principal-component plane (PC1 vs PC2) showing subjects who were reassigned to a different cluster after removal of the disease/smoking dummy variables (*n* = 24, circled in black). Point color denotes the original cluster (blue, High Function; orange, Low Muscle; green, Low Lung/Mobility; red, Severe Impairment). Reassigned subjects lie predominantly along the boundaries between adjacent clusters rather than within cluster cores, confirming that the dummy variables affect peripheral assignments rather than the overall structure.

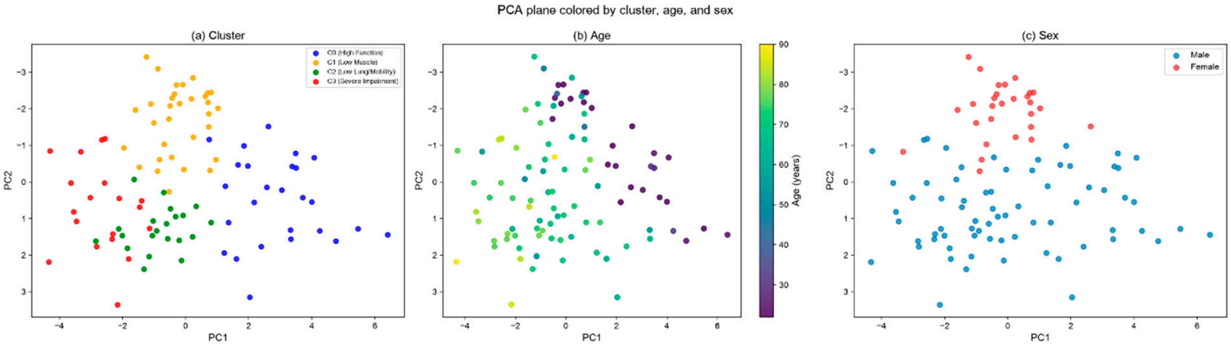

Figure S8. Principal-component plane (PC1 vs PC2) coloured by (a) cluster, (b) age, and (c) sex (*n* = 98). PC1 represents the physical-function axis. Younger and male subjects concentrate in the High-Function region (high PC1), reflecting the age and sex imbalance across clusters (Table S10). This figure illustrates the potential confounding that motivated the age- and sex-adjusted analyses (Tables S11–S14).

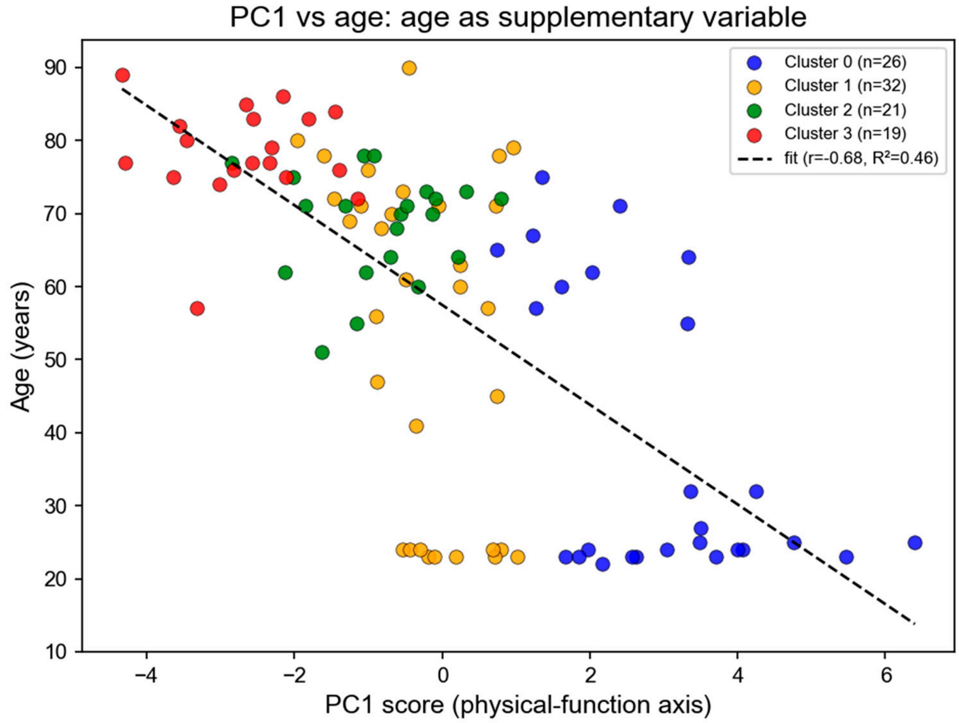

Figure S9. Scatter plot of PC1 score (physical-function axis) versus age, treating age as a supplementary variable external to the PCA (*n* = 98). Points are coloured by cluster. The dashed line is the linear fit (Pearson r = −0.68, R^2 = 0.46), indicating a moderate-to-strong negative correlation between physical function and age. Despite this association, age was not an independent predictor of cluster membership after adjustment (Tables S11-14), consistent with age operating indirectly through reduced physical function.

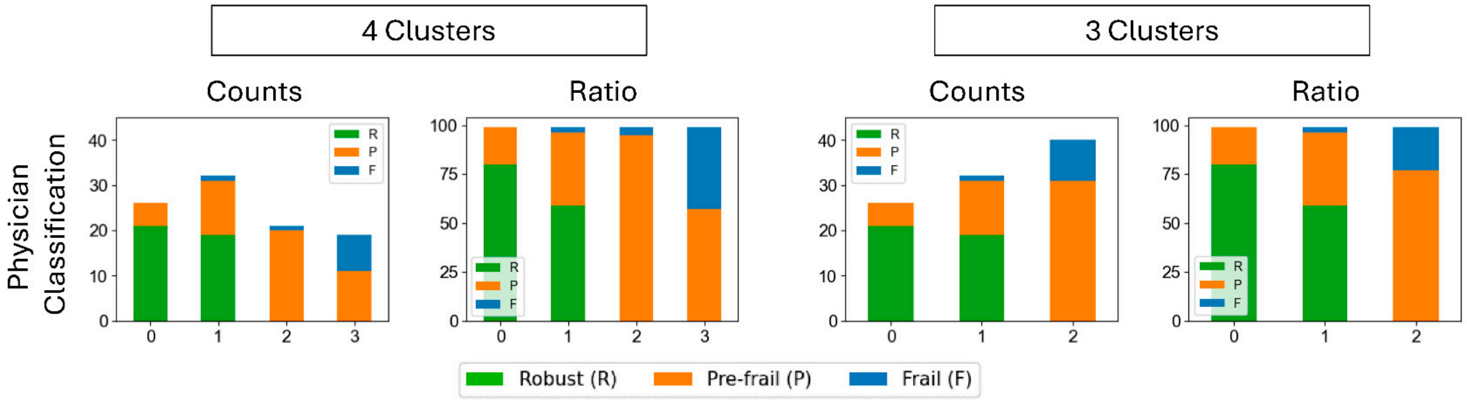

Figure S10. The compositions of three indices (Physician Classification) within each cluster for the baseline measurement. The exact number of subjects is displayed by counts and ratios. The left panels represent the 4-cluster configuration, and the right panels represent the 3-cluster configuration. For all three indices, R to robust, P to pre-frail, and F to Frail**.**

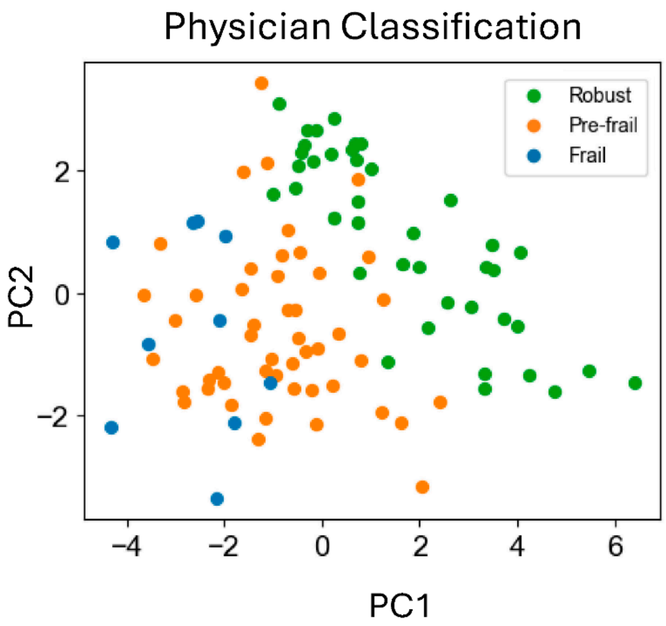

Figure S11. Scatter plots of data points projected onto a plane spanned by PC1 and PC2 from PCA for baseline data measurement. The data points are color-coded according to Physician Classification. The scatter plot demonstrates the distribution of data points corresponding to different levels of each index. The distribution patterns indicate the correspondence between each index's levels.

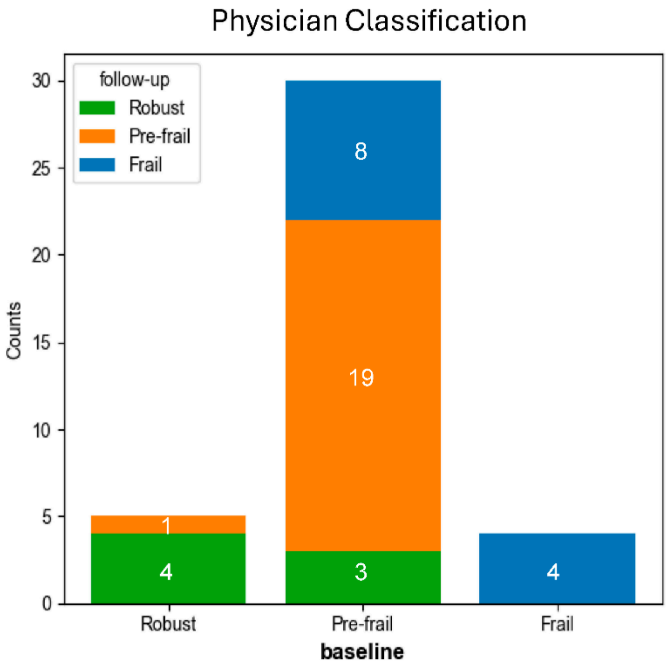

Figure S12. Transitions of indices from baseline to follow-up: Physician Classification. The horizontal axis represents the levels of each index at baseline, and the vertical axis represents the count of individuals. Each bar is stacked to show the distribution of follow-up levels, with numbers indicating the count of individuals. This figure includes data from 39 individuals measured at both time points, highlighting the transitions between measurements.

Supplementary Tables

Table S1. Comparison of clustering methods by agreement with the reference Ward solution (k = 4).

Method	No. of clusters	No. of noise points	ARI vs Ward solution	Parameters
Ward (reference)	4	0	1.000	Euclidean, k = 4
k-means (k=4)	4	0	0.457	k = 4, n_init = 10
GMM (k=4)	4	0	0.369	k = 4, full cov, n_init = 5
DBSCAN (no valid split)	0	98	−	min_samples = 4

k-means (ARI = 0.46) and a Gaussian mixture model (ARI = 0.37) showed moderate agreement, whereas DBSCAN produced no valid multi-cluster split (all 98 subjects assigned to a single group/noise across all eps values), indicating the absence of density gaps and supporting a continuous data structure.

Table S2. Stability of k-means clustering across 50 random initializations (k = 4) under two settings (n_init = 10, the production setting; n_init = 1).

Setting	No. of seeds	Pairwise ARI, mean	Pairwise ARI, minimum	Pairwise ARI, maximum	Identical solution pairs, %	ARI vs Ward, mean	ARI vs Ward, minimum	ARI vs Ward, maximum	No. of distinct solutions	Mean consensus agreement
**n_init=10**	**50**	**0.811**	**0.464**	**1.000**	**3.800**	**0.534**	**0.457**	**0.796**	**26**	**0.935**
**n_init=1**	**50**	**0.598**	**0.228**	**1.000**	**0.200**	**0.576**	**0.241**	**0.800**	**47**	**0.804**

Columns report the number of seeds, mean/min/max pairwise ARI between solutions, percentage of identical solution pairs, mean/min/max ARI versus Ward, number of distinct inertia solutions, and mean consensus agreement. Even with n_init = 10, 26/50 distinct local optima were found and only 3.8% of solution pairs were identical, confirming that k-means does not converge to a unique partition.

Table S3. DBSCAN eps search using k-distance quantiles (min_samples = 4) on the 17-variable standardized data.

ε (eps)	Min. samples	No. of clusters	No. of noise points	ARI vs Ward solution (non-noise points only)
2.455	4	1	29	−
2.539	4	1	22	−
2.688	4	1	16	−
2.713	4	1	12	−
2.850	4	1	6	−
3.031	4	1	4	−
3.145	4	1	3	−
3.269	4	1	1	−

Across all candidate eps values (2.455–3.269) DBSCAN returned a single cluster with a varying number of noise points and never recovered a four-cluster structure, so no ARI could be computed; this indicates no natural density-based separation in the data. ε (eps): DBSCAN neighborhood radius.

Table S6. Omnibus between-cluster differences per variable (*n* = 98).

Variable	ANOVA F	ANOVA p-value	η²	η², 95% CI lower	η², 95% CI upper	ω²	Kruskal–Wallis H	Kruskal–Wallis p-value	ε²	BH-corrected ANOVA p-value	Significant after BH correction
Rt HS	76.429	0.000	0.709	0.600	0.767	0.698	68.552	0.000	0.707	0.000	Yes
6min WT	70.132	0.000	0.691	0.577	0.753	0.679	62.139	0.000	0.641	0.000	Yes
Lt HS	56.848	0.000	0.645	0.517	0.715	0.631	62.836	0.000	0.648	0.000	Yes
WBPhA	38.794	0.000	0.553	0.405	0.639	0.536	53.165	0.000	0.548	0.000	Yes
5m WT	26.095	0.000	0.454	0.292	0.555	0.434	46.055	0.000	0.475	0.000	Yes
%FEV_1	25.907	0.000	0.453	0.290	0.554	0.433	46.701	0.000	0.481	0.000	Yes
FEV_1%	23.679	0.000	0.430	0.266	0.535	0.410	45.490	0.000	0.469	0.000	Yes
Lt LLS	22.665	0.000	0.420	0.255	0.525	0.399	36.067	0.000	0.372	0.000	Yes
Rt LLS	20.647	0.000	0.397	0.232	0.505	0.376	34.293	0.000	0.354	0.000	Yes
SMI	18.078	0.000	0.366	0.200	0.477	0.343	36.269	0.000	0.374	0.000	Yes
%VC	3.477	0.019	0.100	0.002	0.203	0.070	7.656	0.054	0.079	0.019	Yes

All variables were significant after correction. Effect sizes were large for grip strength (right $\eta^2 = 0.71$, left 0.65), 6-min walking speed (0.69), and whole-body phase angle (0.55), and small for %VC ($\eta^2 = 0.10$; note KW $p = 0.054$, the only non-significant nonparametric result). This table is the source for the forest plot in Figure 7.

Table S7. Internal validation metrics for Ward hierarchical clustering across candidate numbers of clusters (k = 2–7; 17 variables (11 standardized + 6 one-hot), *n* = 98).

No. of clusters (k)	Silhouette score	Davies–Bouldin index	Calinski–Harabasz index	Gap statistic	SE of gap
2	0.255	1.413	34.756	0.797	0.023
3	0.211	1.477	33.404	0.952	0.032
4	0.187	1.708	26.905	0.960	0.036
5	0.172	1.736	24.257	1.004	0.032
6	0.150	1.794	21.844	1.029	0.029
7	0.160	1.658	20.297	1.037	0.033

The cophenetic correlation coefficient for the full dendrogram was r = 0.585. By the gap 1-SE criterion the optimal value is k = 3; the four-cluster solution was selected for clinical interpretability and cluster-core stability (see Table S8, Figure S5)

Table S8. Consensus membership by cluster (k = 4).

Cluster (main 4-cluster label)	Mean consensus membership	SD	Minimum	Maximum	n
0	0.654	0.138	0.228	0.766	26
1	0.714	0.127	0.364	0.807	32
2	0.709	0.100	0.510	0.812	21
3	0.865	0.025	0.804	0.898	19

For each cluster (main 4-cluster labels 0–3), the table gives the mean, standard deviation, minimum, maximum, and number of subjects of the within-cluster consensus index (higher mean = more stable core). The Severe-Impairment cluster (3) had the most stable core (0.865 ± 0.025), followed by Low Muscle (0.714), Low Lung/Mobility (0.709), and High Function (0.654), the last showing the widest spread (min 0.228).

Table S9. Physical-function profile of the clusters obtained after removing the disease/smoking dummy variables (observed values; mean ± SD), ordered from robust to frail.

Cluster	%VC	$\%FEV_1$	$FEV_1\%$	Rt HS	Lt HS	5m WT	6min WT	Rt LLS	Lt LLS	WBPhA	SMI	n	Disease [H/nonCOPD/COPD]	Smoking [Non/Ex/Cur]	Female %	Severity score
1	105.0 ± 13.9	100.4 ± 11.2	81.9 ± 12.4	45.9 ± 7.0	42.3 ± 7.2	85.9 ± 9.1	80.1 ± 7.4	51.0 ± 21.0	50.0 ± 20.1	6.7 ± 1.0	7.5 ± 0.7	26	17/4/5	16/6/4	3.8	0.895
2	99.3 ± 12.7	98.6 ± 11.1	82.1 ± 10.9	24.0 ± 4.6	22.3 ± 3.2	82.8 ± 12.3	74.5 ± 7.1	29.8 ± 11.4	27.7 ± 8.5	5.4 ± 0.7	6.0 ± 0.6	25	18/5/2	22/3/0	88.0	-0.121
4	102.0 ± 13.8	81.2 ± 12.6	63.8 ± 12.1	32.1 ± 5.7	30.6 ± 5.5	66.9 ± 17.9	62.8 ± 15.1	30.0 ± 9.9	27.4 ± 9.9	5.0 ± 0.6	7.1 ± 0.8	31	2/8/21	4/23/4	12.9	-0.255
3	80.1 ± 13.8	60.5 ± 19.3	59.3 ± 15.4	27.9 ± 6.8	28.3 ± 7.7	64.2 ± 13.2	52.6 ± 10.5	17.9 ± 6.4	16.5 ± 6.1	4.6 ± 0.9	6.5 ± 1.2	16	0/3/13	0/13/3	6.2	-0.772

Cluster numbering in this table (1–4) is independent of the main four-cluster labels (0–3); the robust-to-frail ordering (rows 1→4) corresponds approximately to High Function → Low Muscle → intermediate → Severe Impairment. The preserved robust-to-frail gradient confirms that cluster structure is driven by physical function rather than by the dummy variables. Higher severity-score values indicate a more robust profile.

Table S10. Age and sex distribution by cluster (*n* = 98).

Cluster	Assigned name	n	Age, mean±SD (years)	Female, n	Female, %
0	High Function	26	38.4 ± 19.5	2	7.70
1	Low Muscle	32	53.5 ± 22.9	25	78.10
2	Low Lung/Mobility	21	68.4 ± 7.3	0	0.00
3	Severe Impairment	19	78.3 ± 6.9	1	5.30

For each cluster the table gives the assigned name, n, age (mean ± SD), number of female subjects, and female percentage. Clusters differed significantly in age (one-way ANOVA $p < 0.001$) and sex (χ^2 $p < 0.001$): High Function was youngest (38.4 ± 19.5 y, 7.7% female) and Severe Impairment oldest (78.3 ± 6.9 y), while Low Muscle was predominantly female (78.1%). These imbalances motivated the adjusted analyses in Tables S11–S14.

Table S11. Ordinal logistic regression of cluster rank on PC1, PC2, age, and sex (continuous predictors standardized; *n* = 98).

Predictor	Coefficient	Odds ratio per 1 SD	SE	p-value
PC1	-5.356	0.005	0.847	0.000
PC2	0.563	1.757	0.427	0.187
age	-0.106	0.899	0.382	0.781
sex	-1.107	0.331	0.933	0.235

PC1 (the physical-function axis) was the only significant predictor (coef = −5.36, OR = 0.0047, $p < 0.001$), whereas age ($p = 0.78$) and sex ($p = 0.24$) were not, indicating that physical function drives cluster membership independently of age and sex.

Table S12. Variance inflation factors (VIF) for the predictors of the ordinal regression model (PC1, PC2, age, sex).

Predictor	VIF
PC1	2.181
PC2	2.898
age	2.522
sex	2.710

All values were below 3 (PC1 = 2.18, PC2 = 2.90, age = 2.52, sex = 2.71), indicating no problematic multicollinearity; the non-significance of age in Table S11 is therefore not attributable to collinearity with the physical-function components.

Table S13. Ordinal-model comparison quantifying the incremental contribution of physical function versus age/sex (*n* = 98).

Model	Predictors	McFadden pseudo-R^2	Log-likelihood	AIC
(a) age + sex	age, sex	0.195	-107.800	225.600
(b) PC1 + PC2 + age + sex (full)	PC1, PC2, age, sex	0.588	-55.090	124.170
(c) PC1 + PC2	PC1, PC2	0.583	-55.800	121.600

Models: (a) age + sex, (b) PC1 + PC2 + age + sex, (c) PC1 + PC2. Columns: McFadden pseudo-R^2, log-likelihood, and AIC. Adding the physical-function components to age + sex increased pseudo-R^2 from 0.195 to 0.588 ($\Delta R^2 = +0.39$), whereas adding age + sex to the PC-only model increased it only from 0.583 to 0.588 ($\Delta R^2 = +0.005$). The PC-only model had the lowest AIC (121.6), indicating that age and sex add essentially no information beyond physical function.

Table S14. Between-cluster differences for each physical-function variable adjusted for age and sex (linear model, type-II ANOVA; *n* = 98).

Variable	Cluster effect, F	p-value for cluster effect (adjusted for age and sex)	BH-corrected p-value
%VC	6.779	0.000	0.000
%FEV_1	14.858	0.000	0.000
FEV_1%	4.482	0.005	0.005
Rt HS	27.321	0.000	0.000
Lt HS	20.313	0.000	0.000
5m WT	16.547	0.000	0.000
6min WT	35.525	0.000	0.000
Rt LLS	6.379	0.001	0.001
Lt LLS	8.277	0.000	0.000
WBPhA	9.674	0.000	0.000
SMI	6.883	0.000	0.000

All 11 variables remained significant after adjustment (BH-corrected $p \leq 0.005$), with the strongest effects for 6min WT ($F = 35.5$), Rt HS ($F = 27.3$), and Lt HS ($F = 20.3$), confirming that cluster differences in physical function are not explained by age or sex confounding.

Table S15. Characteristics of the study participants (J-CHS).

	J-CHS			
Characteristics	Frail (*n*=9)	Pre-frail (*n*=49)	Robust (*n*=40)	*p*-value
Age (years)	72.1 ± 7.7	63.9 ± 19.1	46.4 ± 23.5	0.0014
Gender (male/female)	8/1	35/14	27/13	0.439
BMI (kg/m^2)	22.2 ± 5.3	23.0 ± 4.0	22.0 ± 2.9	0.664
SMI (kg/m^2)	6.7 ± 1.2	6.7 ± 1.0	7.0 ± 1.0	0.422
WBPhA (°)	4.7 ± 1.1	5.2 ± 0.8	6.0 ± 1.3	0.003
Rt HS (kg)	25.8 ± 6.6	31.3 ± 7.9	36.7 ± 12.1	0.014
Lt HS (kg)	22.4 ± 4.1	30.2 ± 7.0	34.4 ± 11.3	0.003
Rt LLS (kg)	18.4 ± 8.0	29.4 ± 9.8	42.1 ± 22.2	0.0003
Lt LLS (kg)	17.4 ± 5.5	26.5 ± 10.0	41.2 ± 20.8	<0.0001
Disease [COPD/Non-COPD/Healthy controls]	17/7/0	18/5/10	6/8/27	<0.0001
Smoking history [Cu/Ex/Non]	2/7/0	3/29/17	6/9/25	0.0006
%VC (%)	87.3 ± 15.7	98.8 ± 17.4	100.7 ± 12.8	0.118
%FEV$_1$ (%)	71.1 ± 15.2	82.6 ± 21.1	96.8 ± 13.0	<0.0001
FEV$_1$% (%)	64.2 ± 10.3	67.9 ± 16.4	80.0 ± 13.1	0.0001
6min WT speed (m/min)	53.7 ± 16.5	65.0 ± 14.3	76.6 ± 9.3	<0.0001
5m WT speed (m/min)	55.5 ± 14.2	71.8 ± 15.0	84.7 ± 12.8	<0.0001

%VC, percentages of predicted vital capacity; FVC, forced vital capacity; %FEV$_1$, percentages of predicted forced expiratory volume in one second; FEV$_1$%, FEV$_1$/FVC%; 6min WT, 6-minute walk test; 5m WT, 5-meter walk test; Rt, right; Lt, left, HS, handgrip strength; LLS, Lower Limb Strength; WBPhA, whole–body phase angle; SMI, skeletal muscle mass index. Data are presented as mean ± standard deviation (STD) unless otherwise stated. Differences between groups were assessed using one-way analysis of variance. Categorical data are compared using Pearson's chi-squared test.

Table S16. Characteristics of the study participants (Kihon Check List).

	Kihon CheckList			
Characteristics	Frail (*n*=24)	Pre-frail (*n*=33)	Robust (*n*=41)	*p*-value
Age (years)	73.5 ± 9.6	61.8 ± 19.4	44.6 ± 22.7	<0.0001
Gender (male/female)	22/2	24/9	24/17	0.0167
BMI (kg/m^2)	23.2 ± 5.1	22.7 ± 3.3	21.9 ± 3.0	0.4750
SMI (kg/m^2)	6.9 ± 1.1	6.7 ± 0.7	6.9 ± 1.1	0.6774
WBPhA (°)	4.8 ± 0.8	5.4 ± 1.1	6.0 ± 1.0	<0.0001
Rt HS (kg)	30.1 ± 7.8	31.8 ± 9.5	35.7 ± 11.6	0.1268
Lt HS (kg)	29.1 ± 7.0	30.5 ± 9.9	33.0 ± 10.1	0.3351
Rt LLS (kg)	25.2 ± 11.1	32.0 ± 16.4	39.6 ± 19.7	0.0126
Lt LLS (kg)	21.4 ± 9.4	30.4 ± 13.9	38.7 ± 19.9	0.0002
Disease [COPD/Non-COPD/Healthy controls]	17/7/0	18/5/10	6/8/27	<0.0001
Smoking history [Cu/Ex/Non]	5/17/2	3/17/13	3/11/27	0.0003
%VC (%)	94.4 ± 19.4	100.2 ± 15.6	99.6 ± 13.5	0.5147
%FEV$_1$ (%)	75.3 ± 19.8	84.4 ± 19.1	96.7 ± 14.8	<0.0001
FEV$_1$% (%)	63.0 ± 13.6	68.5 ± 17.0	81.3 ± 11.2	<0.0001
6min WT speed (m/min)	56.9 ± 17.2	67.8 ± 12.5	76.3 ± 8.7	<0.0001
5m WT speed (m/min)	64.2 ± 16.9	74.6 ± 15.2	82.9 ± 13.3	0.0001

%VC, percentages of predicted vital capacity; FVC, forced vital capacity; %FEV$_1$, percentages of predicted forced expiratory volume in one second; FEV$_1$%, FEV$_1$/FVC%; 6min WT, 6-minute walk test; 5m WT, 5-meter walk test; Rt, right; Lt, left, HS, handgrip strength; LLS, Lower Limb Strength; WBPhA, whole–body phase angle; SMI, skeletal muscle mass index. Data are presented as mean ± standard deviation (STD) unless otherwise stated. Differences between groups are assessed using one-way analysis of variance. Categorical data are compared using Pearson's chi-squared test.

Table S17. Characteristics of the study participants (Physician classification).

	Physician classification			
Characteristics	Frail (*n*=10)	Pre-frail (*n*=48)	Robust (*n*=40)	*p*-value
Age (years)	81.8 ± 4.3	70.2 ± 7.9	36.1 ± 18.5	<0.0001
Gender (male/female)	10/0	40/8	20/20	0.0003
BMI (kg/m^2)	22.6 ± 5.1	23.2 ± 4.0	21.7 ± 2.7	0.206
SMI (kg/m^2)	6.7 ± 1.1	6.9 ± 0.9	6.8 ± 1.0	0.451
WBPhA (°)	4.2 ± 0.7	5.1 ± 0.7	6.3 ± 1.1	<0.0001
Rt HS (kg)	26.2 ± 7.7	32.2 ± 8.4	35.7 ± 12.0	0.060
Lt HS (kg)	26.2 ± 4.8	31.1 ± 8.5	32.6 ± 11.0	0.246
Rt LLS (kg)	23.7 ± 9.4	27.9 ± 11.6	42.8 ± 21.1	0.0003
Lt LLS (kg)	16.5 ± 6.1	26.8 ± 11.0	41.3 ± 20.0	<0.0001
Disease [COPD/Non-COPD/ Healthy controls]	7/3/0	33/12/3	1/5/34	<0.0001
Smoking history [Cu/Ex/Non]	1/9/0	9/31/8	1/5/34	<0.0001
%VC (%)	96.5 ± 20.6	98.6 ± 17.2	99.0 ± 12.8	0.864
%FEV$_1$ (%)	75.2 ± 19.8	80.2 ± 19.7	99.0 ± 12.0	<0.0001
FEV$_1$% (%)	62.5 ± 18.9	63.4 ± 10.9	86.0 ± 9.3	<0.0001
6min WT speed (m/min)	44.5 ± 13.6	64.2 ± 10.7	80.1 ± 6.0	<0.0001
5m WT speed (m/min)	59.5 ± 13.5	71.1 ± 17.2	84.9 ± 10.0	<0.0001

%VC, percentages of predicted vital capacity; FVC, forced vital capacity; %FEV$_1$, percentages of predicted forced expiratory volume in one second; FEV$_1$%, FEV$_1$/FVC%; 6min WT, 6-minute walk test; 5m WT, 5-meter walk test; Rt, right; Lt, left, HS, handgrip strength; LLS, Lower Limb Strength; WBPhA, whole–body phase angle; SMI, skeletal muscle mass index. Data are presented as mean ± standard deviation (STD) unless otherwise stated. Differences between groups were assessed using one-way analysis of variance. Categorical data are compared using Pearson’s chi-squared test.

Table S18. Summary of the study participants' diseases.

	Class all (*n*=98)	COPD (*n*=41)	Non-COPD (*n*=20)	Robust (*n*=37)	*p*-value
	mean ± STD	mean ± STD	mean ± STD	mean ± STD	
%VC (%)	98.5 ± 15.8	100.6 ± 19.2	96.1 ± 14.3	97.5 ± 12.1	0.519
$\%FEV_1$ (%)	87.3 ± 19.5	75.5 ± 18.6	89.0 ± 17.7	99.5 ± 12.7	<0.0001
$FEV_1\%$ (%)	72.5 ± 15.8	59.0 ± 11.2	71.7 ± 8.4	87.9 ± 7.1	<0.0001
5m WT speed (m/min)	75.5 ± 16.5	65.8 ± 15.0	77.1 ± 17.3	85.5 ±10.7	<0.0001
6min WT speed (m/min)	68.7 ± 14.6	59.5 ± 13.5	68.9 ± 15.2	78.8 ± 6.8	<0.0001
Rt HS (kg)	33.0 ± 10.3	32.7 ± 8.4	30.3 ± 8.4	34.9 ± 12.6	0.268
Lt HS (kg)	31.2 ± 9.4	31.1 ± 8.3	30.1 ± 7.3	32.0 ± 11.6	0.773
Rt LLS (kg)	33.5 ± 17.7	28.0 ± 11.6	32.0 ± 17.1	40.5 ± 21.3	0.0062
Lt LLS (kg)	31.7 ± 17.2	25.2 ± 11.3	31.6 ± 18.5	38.9 ± 19.3	0.0015
WBPhA (°)	5.5 ± 1.1	5.0 ± 0.8	5.0 ± 0.9	6.3 ± 1.1	<0.0001
SMI (kg/m^2)	6.8 ± 1.0	7.0 ± 0.9	7.0 ± 1.1	6.6 ± 1.0	0.190

%VC, percentages of predicted vital capacity; FVC, forced vital capacity; $\%FEV_1$, percentages of predicted forced expiratory volume in one second; $FEV_1\%$, $FEV_1/FVC\%$; 6min WT, 6-minute walk test; 5m WT, 5-meter walk test; Rt, right; Lt, left, HS, handgrip strength; LLS, Lower Limb Strength; WBPhA, whole–body phase angle; SMI, skeletal muscle mass index. Data are presented as mean ± standard deviation (STD). Differences between groups are assessed using one-way analysis of variance.

Table S19. Adjusted Rand Index (ARI) for clusters 3, 4, and 5 against J-CHS, KCL and Physician Classification.

	3 clusters	4 clusters	5 clusters
J-CHS	0.16	0.14	0.10
KCL	0.18	0.16	0.11
Physician Classification	0.27	0.22	0.18

CHS, Cardiovascular Health Study; KCL, Kihon Checklist

Table S20. Summary of all parameters measured at baseline data collection (three clusters).

	Class all (n=98)	Class 0 (n=26)	Class1 (n=32)	Class2 (n=40)
	mean ± STD	mean ± STD	mean ± STD	mean ± STD
%VC (%)	98.5 ± 15.8	105.7 ± 14.3	97.3 ± 13.0	94.9 ± 17.5
%FEV_1 (%)	87.3 ± 19.5	100.6 ± 11.4	95.9 ± 12.1	71.8 ± 17.9
FEV_1% (%)	72.5 ± 15.8	81.7 ± 12.5	80.6 ± 10.5	60.1 ± 13.0
5m WT speed (m/min)	75.5 ± 16.5	85.8 ± 9.2	82.8 ± 13.4	63.1 ± 14.5
6min WT speed (m/min)	68.7 ± 14.6	79.7 ± 7.3	75.0 ± 7.2	56.5 ± 13.7
Rt HS (kg)	33.0 ± 10.3	46.0 ± 6.8	25.1 ± 5.0	31.0 ± 6.6
Lt HS (kg)	31.2 ± 9.4	42.1 ± 7.5	23.7 ± 4.5	30.2 ± 6.5
Rt LLS (kg)	33.5 ± 17.7	51.3 ± 20.8	28.5 ± 10.5	26.0 ± 11.0
Lt LLS (kg)	31.7 ± 17.2	49.3 ± 20.6	28.1 ± 9.1	23.0 ± 10.1
WBPhA (°)	5.5 ± 1.1	6.8 ± 1.0	5.3 ± 0.7	4.8 ± 0.7
SMI (kg/m^2)	6.8 ± 1.0	7.5 ± 0.8	6.2 ± 0.7	7.0 ± 1.0
smoke 0 (Non-smoker)	0.4 ± 0.5	0.7 ± 0.5	0.8 ± 0.4	0.0 ± 0.2
smoke 1 (Ex-smoker)	0.5 ± 0.5	0.2 ± 0.4	0.2 ± 0.4	0.8 ± 0.4
smoke 2 (Current smoker)	0.1 ± 0.3	0.1 ± 0.3	0.1 ± 0.2	0.2 ± 0.4
disease 0 (Healthy controls)	0.4 ± 0.5	0.7 ± 0.5	0.6 ± 0.5	0.0 ± 0.0
disease 1 (Non-COPD)	0.2 ± 0.4	0.1 ± 0.3	0.3 ± 0.5	0.2 ± 0.4
disease 2 (COPD)	0.4 ± 0.5	0.2 ± 0.4	0.1 ± 0.3	0.8 ± 0.4

%VC, percentages of predicted vital capacity; FVC, forced vital capacity; %FEV_1, percentages of predicted forced expiratory volume in one second; FEV_1%, FEV_1/FVC%; 6min WT, 6-minute walk test; 5m WT, 5-meter walk test; Rt, right; Lt, left, HS, handgrip strength; LLS, Lower Limb Strength; WBPhA, whole–body phase angle; SMI, skeletal muscle mass index. Data are presented as mean ± standard deviation (STD) unless otherwise stated.

Table S21. Summary of all parameters measured at follow-up data collection.

	Class all (n=39)	Class 0 (n=2)	Class1 (n=8)	Class2 (n=19)	Class3 (n=10)
	mean ± std	mean ± std	mean ± std	mean ± std	mean ± std
%VC (%)	99.2 ± 21.3	102.8 ± 9.7	106.8 ± 16.5	100.3 ± 16.4	90.2 ± 31.7
%FEV_1 (%)	80.4 ± 20.2	95.5 ± 2.5	98.3 ± 15.5	77.3 ± 14.1	68.7 ± 25.2
FEV_1% (%)	63.8 ± 12.7	73.8 ± 1.2	70.1 ± 6.1	61.4 ± 7.3	61.5 ± 21.6
5m WT speed (m/min)	73.6 ± 18.0	91.9 ± 1.8	89.8 ± 19.5	74.6 ± 10.4	54.9 ± 12.4
6min WT speed (m/min)	65.1 ± 15.7	86.3 ± 4.7	73.3 ± 9.6	68.6 ± 8.6	47.8 ± 17.5
Rt HS (kg)	31.0 ± 7.6	43.2 ± 2.3	27.4 ± 4.8	34.6 ± 6.8	24.6 ± 4.0
Lt HS (kg)	29.3 ± 8.2	41.2 ± 2.5	26.8 ± 4.8	32.6 ± 7.6	22.4 ± 6.4
Rt LLS (kg)	27.7 ± 13.3	51.2 ± 1.1	29.5 ± 12.9	30.4 ± 11.6	16.3 ± 8.2
Lt LLS (kg)	27.2 ± 12.7	50.8 ± 1.8	31.2 ± 13.5	28.6 ± 11.0	16.5 ± 5.3
WBPhA (°)	4.9 ± 0.7	6.0 ± 0.3	5.0 ± 0.5	5.2 ± 0.6	4.2 ± 0.5
SMI (kg/m^2)	6.9 ± 0.8	8.2 ± 0.7	6.7 ± 0.8	7.2 ± 0.6	6.0 ± 0.6
Non-smoker	0.1 ± 0.3	0.0 ± 0.0	0.6 ± 0.5	0.0 ± 0.0	0.0 ± 0.0
Ex-smoker	0.7 ± 0.5	1.0 ± 0.0	0.2 ± 0.5	0.8 ± 0.4	0.7 ± 0.5
Current smoker	0.2 ± 0.4	0.0 ± 0.0	0.1 ± 0.4	0.2 ± 0.4	0.3 ± 0.5
Healthy controls	0.0 ± 0.0	0.0 ± 0.0	0.0 ± 0.0	0.0 ± 0.0	0.0 ± 0.0
Non COPD	0.3 ± 0.5	1.0 ± 0.0	0.9 ± 0.4	0.2 ± 0.4	0.0 ± 0.0
COPD	0.7 ± 0.5	0.0 ± 0.0	0.1 ± 0.4	0.8 ± 0.4	1.0 ± 0.0

%VC, percentages of predicted vital capacity; FVC, forced vital capacity; %FEV_1, percentages of predicted forced expiratory volume in one second; FEV_1%, FEV_1/FVC%; 6min WT, 6-minute walk test; 5m WT, 5-meter walk test; Rt, right; Lt, left, HS, handgrip strength; LLS, Lower Limb Strength; WBPhA, whole–body phase angle; SMI, skeletal muscle mass index. Data are mean ± std unless otherwise stated. Differences between groups were assessed using a one-way ANOVA. Categorical data were compared using Pearson's chi-squared test.
